# Advances in heavy alkaline earth chemistry provide insight into complexation of weakly polarizing Ra^2+^, Ba^2+^, and Sr^2+^ cations

**DOI:** 10.1126/sciadv.adj8765

**Published:** 2024-01-05

**Authors:** J. Connor Gilhula, Lei Xu, Frankie D. White, Sara L. Adelman, Kelly E. Aldrich, Enrique R. Batista, David Dan, Zachary R. Jones, Stosh A. Kozimor, Harris E. Mason, Rachel L. Meyer, Nikki A. Thiele, Ping Yang, Mingbin Yuan

**Affiliations:** ^1^ Los Alamos National Laboratory, Los Alamos, NM 87545 (USA).; ^2^ Department of Chemistry, University of Rochester, Rochester, NY 14627 (USA).; ^3^ Chemical Sciences Division, Oak Ridge National Laboratory, Oak Ridge, TN 37831 (USA).

## Abstract

Numerous technologies—with catalytic, therapeutic, and diagnostic applications—would benefit from improved chelation strategies for heavy alkaline earth elements: Ra^2+^, Ba^2+^, and Sr^2+^. Unfortunately, chelating these metals is challenging because of their large size and weak polarizing power. We found 18-crown-6-tetracarboxylic acid (**H**_**4**_**COCO**) bound Ra^2+^, Ba^2+^, and Sr^2+^ to form **M(H**_***x***_**COCO)**^***x*–2**^. Upon isolating radioactive ^223^Ra from its parent radionuclides (^227^Ac and ^227^Th), ^223^Ra^2+^ reacted with the fully deprotonated **COCO**^**4−**^ chelator to generate **Ra(COCO)**^**2−**^_**(*aq*)**_ (log *K*_**Ra(COCO)2−**_ = 5.97 ± 0.01), a rare example of a molecular radium complex. Comparative analyses with Sr^2+^ and Ba^2+^ congeners informed on what attributes engendered success in heavy alkaline earth complexation. Chelators with high negative charge [−4 for **Ra(COCO)**^**2−**^_**(*aq*)**_] and many donor atoms [≥11 in **Ra(COCO)**^**2−**^_**(*aq*)**_] provided a framework for stable complex formation. These conditions achieved steric saturation and overcame the weak polarization powers associated with these large dicationic metals.

## Teaser

Chelation of weakly polarizing and sterically demanding metals was achieved by studying **COCO**^**4−**^ binding of Ra^2+^, Ba^2+^, and Sr^2+^.

## INTRODUCTION

Improved understanding of heavy alkaline earth element (Ra^2+^, Ba^2+^, and Sr^2+^) complexation chemistry is challenging current assumptions about the chemistry of these elements and opening doors into new application spaces (*1*). These applications range from developing molecular Ba^2+^ and Sr^2+^ catalysts (*2*, *3*) to usage of Ra^2+^ (*4*–*9*), Ba^2+^ (*10*, *11*), and Sr^2+^ (*12*, *13*) in the medical field. A prime example of this notion is Wu, Zhou, Frenking, and coworkers’ recent discovery of the octakis-carbonyl complexes of barium and strontium, M(CO)_8_ (M = Ba and Sr) (*14*–*16*). Identifying experimental conditions that enabled complexation of barium and strontium by CO initiated a changing viewpoint within the chemistry community. Many are leaving the opinion that heavy alkaline earth elements are simple metals with a +2 charge that behave exclusively as Lewis acids. Instead, there is an evolving perspective that these elements can exhibit diverse transition metal–like reactivity (*1*, *14*–*16*).

Armed with that insight, researchers are beginning to use Ra^2+^, Ba^2+^, and Sr^2+^ in ways that historically have been reserved for *d*-block transition elements. For instance, Ba^2+^ and Sr^2+^ complexes are emerging as effective catalysts for a wide range of reaction types, such as imine (*17*) and olefin (*18*–*20*) hydrogenation, hydroaminations (*21*–*25*), dehydrocouplings (*26*–*28*), and polymerizations (*29*–*31*), among others (*32*–*36*). Other applications include emerging use of Ra^2+^, Ba^2+^, and Sr^2+^ in the medical field as diagnostics and therapeutics: examples are alpha therapy (^223^Ra and ^224^Ra) (*4*–*9*), single-photon emission computed tomography (^131^Ba and ^135m^Ba) (*10*), and radiography (^nat^Ba, ^85^Sr, and ^87m^Sr) (*11*–*13*). All of the aforementioned heavy alkaline earth chemistry would benefit from advancing chelation strategies and complexation methodology for Ra^2+^, Ba^2+^, and Sr^2+^.

A major chemical obstacle facing the development of heavy alkaline earth technologies is designing appropriate ligands that irreversibly bind Ra^2+^, Ba^2+^, and Sr^2+^ under relevant experimental conditions. Chelating these metals represents a formidable challenge, potentially more difficult than complexing 3*d*-, 4*d*-, and 5*d*-transition elements, lanthanides, and actinides. For instance, ionicity (as opposed to covalency) is assumed to be the primary driver for bond formation in heavy alkaline earth compounds (*37*). The Ra^2+^, Ba^2+^, and Sr^2+^ dications are also quite large. The 1.7-Å ionic radius of Ra^2+^ (12 coordinate) is greater than 97% of the 498 entries in the Shannon ionic radii tables published in 1976 (*38*). For calibration, the Ra^2+^ dication is substantially larger than +3 and +2 lanthanide and actinide elements and only slightly smaller than the biggest +1 alkali metal monocations (Fr^1+^, Cs^1+^, and Rb^1+^). As a result, the heavy alkaline earths’ +2 charge is distributed over a very large atomic sphere, making these metals weakly Lewis acidic and weakly polarizing. Note that polarizing power informs on the ability to deform a complementary anion and is proportional to Z/r^2^ (Z = ionic charge; r = ionic radius) (*39*). The large ionic radii additionally necessitate high coordination numbers to reach steric saturation. These properties make it difficult to (i) design a chelator that can bind Ra^2+^, Ba^2+^, and Sr^2+^ strongly and (ii) identify experimental conditions for complexing heavy alkaline earth elements. Another complication in heavy alkaline earth chelation chemistry is radioactivity associated with radium. All isotopes of radium are radioactive, and medically relevant isotopes have short half-lives on the order of days (*40*). This property makes it more difficult to obtain and safely handle large quantities of radium for coordination chemistry studies than for stable elements.

**Fig. 1.**
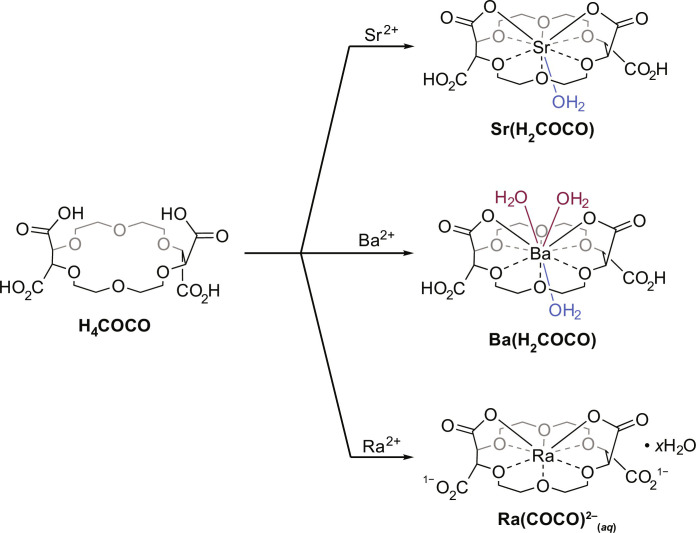
Complexation of Ra^2+^, Ba^2+^, and Sr^2+^ by H_4_COCO. **Sr(H**_**2**_**COCO)** and **Ba(H**_**2**_**COCO)** were synthesized by mixing **H**_**4**_**COCO** with 1 equivalent of Sr(NO_3_)_2_ or Ba(NO_3_)_2_, respectively, in a solution of ethanol and water (1:1 EtOH:H_2_O). **Ra(COCO)**^**2−**^_**(*aq*)**_ was generated by dissolving ^223^Ra^2+^_(*aq*)_ in aqueous solution (either unbuffered or buffered with MES at pH 6.29) containing excess **COCO**^**4−**^_**(*aq*)**_.

Motivated by the fundamental challenge to chelate heavy alkaline earth metals and excited to support future innovation in Ra^2+^, Ba^2+^, and Sr^2+^ technologies through expansion of their chelation chemistry, we launched a study focused on binding these ions with a macrocyclic crown ether derivative, (−)-(18-crown-6)-2,3,11,12-tetracarboxylic acid (**H**_**4**_**COCO**; Fig. 1) (*41*). We selected this chelator because of its large number of potential biding sites (10 total), high negative charge (−4 when all carboxylic acid functions are deprotonated), and successful complexation studies reported previously with alkaline earth cations (*42*–*44*). It was not possible to monitor Ra^2+^_(*aq*)_ binding by **H**_**4**_**COCO**_**(*aq*)**_ through spectroscopic methods commonly used to characterize coordination compounds, owing to the small quantities of radium available in our laboratory. To overcome these issues, we isolated 7.8 × 10^5^ Bq (21 μCi, 4.1 × 10^−7^ mg, 1.1 × 10^12^ atoms) of the short-lived ^223^Ra radionuclide [half-life, *t*_½_ = 11.43(5) d] (*45*) from an ^227^Ac source. We then developed a synthetic procedure to prepare **Ra(COCO)**^**2−**^_**(*aq*)**_ and characterized this complex using radiochemical assays. The resulting **Ra(COCO)**^**2−**^_**(*aq*)**_ compound represents a rare example of a molecular complex of radium.

Additional insight into the **Ra(COCO)**^**2**−^_**(*aq*)**_ coordination complex was inferred by characterizing reactivity between congeners of radium (Ba^2+^ and Sr^2+^) and the same **H**_**4**_**COCO** complexing agent. Traditional characterization methods were used for these studies because barium and strontium are naturally occurring and can be obtained in large quantities. That is, **Ba(H**_**2**_**COCO)** and **Sr(H**_**2**_**COCO)** were characterized by single-crystal x-ray diffraction (XRD), solution-phase nuclear magnetic resonance (NMR) spectroscopy, and high-resolution mass spectrometry (HRMS). We also leveraged density functional theory (DFT) calculations to further explicate **Ba(H**_**2**_**COCO)** molecular fluxionality in CD_3_CN solution. The results demonstrated that decreasing the metal polarization power (and Lewis acidity) and increasing the ionic radius of the alkaline earth dication had substantial impact on M^2+^ complexation. These factors influenced Sr^2+^ versus Ba^2+^ coordination numbers, the fluxional processes available to **M(H**_**2**_**COCO)** in solution, and how **H**_**2**_**COCO**^**2−**^ held M^2+^ in its metal binding pocket. Those data can be reasonably extrapolated toward Ra^2+^ and provided a framework to rationalize complexation of Ra^2+^_(*aq*)_ by **H**_**4**_**COCO**_**(*aq*)**_. Overall, this work advances understanding of the structure and coordination chemistry of alkaline earth complexes and provides insight to the salient features of Ra^2+^, Ba^2+^, and Sr^2+^ binding that make heavy alkaline earth chelation chemistry of interest from a fundamental perspective.

## RESULTS

### Isolation and complexation of ^223^Ra^2+^_(*aq*)_

**Fig. 2.**
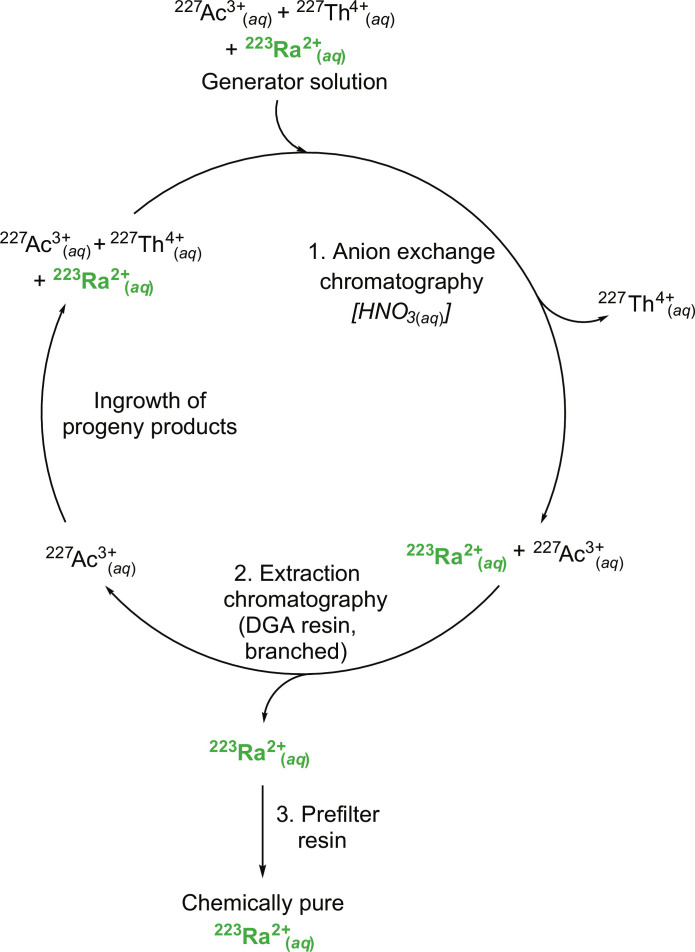
Isolation of ^223^Ra^2+^_(*aq*)_. ^223^Ra^2+^_(*aq*)_ was separated from ^227^Ac^3+^_(*aq*)_ and ^227^Th^4+^_(*aq*)_ in three steps to provide ^223^Ra^2+^_(*aq*)_ in high chemical and radiochemical purity.

Radiochemical methods were used to obtain ^223^Ra and study its complexation chemistry with the **H**_**4**_**COCO** chelator. Success relied on harvesting ^223^Ra^2+^_(*aq*)_ from a solution that contained the ^227^Ac^3+^_(*aq*)_ and ^227^Th^4+^_(*aq*)_ parent radionuclides. This was achieved using a three-step procedure (Fig. 2) that was adapted from previously published methods (*46*, *47*) used to separate Ac^3+^_(*aq*)_ from Th^4+^_(*aq*)_ and Ra^2+^_(*aq*)_. In step one, ^227^Th^4+^_(*aq*)_ was separated from the ^227^Ac^3+^_(*aq*)_ and ^223^Ra^2+^_(*aq*)_ radionuclides using anion exchange chromatography. In step two, extraction chromatography was used to separate ^223^Ra^2+^_(*aq*)_ from ^227^Ac^3+^_(*aq*)_. Last, in step three, organic contaminants introduced from the anion and extraction chromatography steps (*46*, *48*, *49*) were removed from ^223^Ra^2+^_(*aq*)_ using Pre-Filter Resin. The ^223^Ra processing method was attractive because it could be completed on a time scale (~2 to 3 days) that was compatible with the relatively fast ^223^Ra decay rate. In addition, this method provided chemically and radiochemically pure stock solutions of ^223^Ra^2+^_(*aq*)_ in a matrix that was suitable for subsequent complexation efforts.

Complexation of ^223^Ra^2+^_(*aq*)_ by **COCO**^**4−**^_**(*aq*)**_ was achieved by mixing minute quantities of ^223^Ra^2+^_(*aq*)_ (2.6 × 10^5^ Bq, 7.0 μCi, 1.4 × 10^−7^ mg, 3.7 × 10^11^ atoms) with excess **COCO**^**4−**^_**(*aq*)**_ (0.1 mg, approximately 370,000 equivalents) in H_2_O for 45 min at room temperature (Fig. 1). We estimated the pH of this unbuffered aqueous solution to be near 3.1 based on the protonation constants in table S1 (see Supplementary Materials). Unreacted (i.e., uncomplexed) ^223^Ra^2+^_(*aq*)_ was separated from the resulting **Ra(COCO)**^**2−**^_**(*aq*)**_ complex by passing the mixture through a column packed with Chelex 100 resin. Chelex 100 is well established for scavenging uncomplexed +2 cations (*50*), including Ra^2+^ (*51*). In a control experiment, 99.2% of ingoing ^223^Ra activity was retained by the resin when pristine solutions of ^223^Ra^2+^_(*aq*)_ [not mixed with **COCO**^**4−**^_**(*aq*)**_] were passed through a Chelex 100 column. In contrast, when solutions that contained a mixture of ^223^Ra^2+^_(*aq*)_ and **COCO**^**4−**^_**(*aq*)**_ were passed through a Chelex 100 column under identical conditions, only 70% of ingoing ^223^Ra activity was retained by the resin. Meanwhile, the remaining 30% of ^223^Ra activity passed through the column. These data indicated that Ra^2+^_(*aq*)_ and **COCO**^**4−**^_**(*aq*)**_ reacted to generate a coordination complex in 30% yield after contact with Chelex 100. It is important to realize that this value represents how much **Ra(COCO)**^**2−**^_**(*aq*)**_ eluted from the column and not necessarily how much Ra^2+^_(*aq*)_ was initially bound by **COCO**^**4−**^_**(*aq*)**_ before the Chelex purification step. It is possible that the iminodiacetate functionality from the Chelex 100 resin removed free Ra^2+^_(*aq*)_ from the mobile phase (as intended) and additionally stripped some Ra^2+^ from the **Ra(COCO)**^**2−**^_**(*aq*)**_ complex, resulting in a lower isolated yield than would otherwise be expected.

**Fig. 3.**
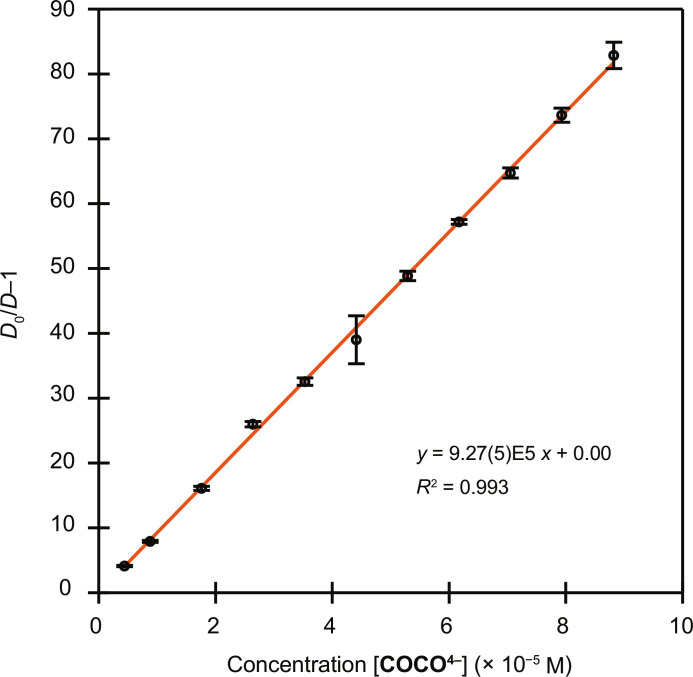
Determination of Ra(COCO)^2−^_(*aq*)_ conditional stability constant. The ratio of distribution ratios of Ra^2+^ when **COCO**^**4−**^_**(*aq*)**_ was absent (*D*_0_) versus present (*D*) in the aqueous phase plotted as a function of **COCO**^**4−**^_**(*aq*)**_ concentration (pH 6.29 with ionic strength set to 0.2 M by NaCl). The data were fit with a line, and the logarithm of the slope from that function, 5.97 (*1*), was log β_app_. The data represent the average of three replicates, and the data point uncertainty was determined as the SD from the mean at 1σ.

Conventional methods used to characterize inorganic coordination complexes (e.g., single-crystal XRD, NMR spectroscopy, and ultraviolet-visible spectroscopy) could not be used to characterize the product(s) from the reaction between ^223^Ra^2+^_(*aq*)_ and **COCO**^**4−**^_**(*aq*)**_ because of the small quantities of ^223^Ra available. Instead, we used radioanalytical methods to probe the speciation and complexation thermodynamics of this system. First, we investigated the protonation states of the free **H**_**4**_**COCO**_**(*aq*)**_ ligand: Potentiometric titrations conducted on **H**_**4**_**COCO**_**(*aq*)**_ indicated that this complexing agent was fully deprotonated in aqueous solutions at pH > 6 and existed as **COCO**^**4−**^_**(*aq*)**_ (see the Supplementary Materials for protonation constant measurements). Next, stability constant measurements between Ra^2+^_(*aq*)_ and this fully deprotonated **COCO**^**4−**^_**(*aq*)**_ ligand were carried out in two steps (*7*, *52*, *53*). In the first step, samples containing cation exchange resin (Dowex 50W X8) and varying equivalents of **COCO**^**4−**^_**(*aq*)**_ were prepared in buffered [2-(*N*-morpholino)ethanesulfonic (MES) acid, ionic strength set to 0.2 M using NaCl] aqueous solution at pH 6.29. Second, each sample was spiked with an aliquot of ^223^Ra^2+^_(*aq*)_. The samples were mixed for 24 hours to allow for thermodynamic equilibrium to be reached, and then the samples were centrifuged. Aliquots of the supernatant were collected and measured by liquid scintillation counting. The distribution coefficient (*D* value) of each sample was subsequently calculated as the ratio of activity adsorbed to the resin versus activity in the aqueous phase at equilibrium as in Eq. 1

$$D=\frac{A_{\text{total}}-A_{\text{aq}}}{A_{\text{aq}}}$$ (1)

where *A*_total_ is the total ingoing activity and *A*_aq_ is the activity that remains in solution after contacting the resin. From the distribution ratios of ^223^Ra in the presence (*D*) and absence (*D*_0_) of varying concentrations of **COCO**^**4−**^_**(*aq*)**_, the conditional cumulative stability constant of metal-ligand complexation (log β_app_) was determined to be 5.97 ± 0.01 (Fig. 3) via linear regression according to Eq. 2

$$\frac{D_{0}}{D}-1=\beta_{\text{app}}\left[{\text{COCO}}^{n–}\right]$$ (2)

**Fig. 4.**
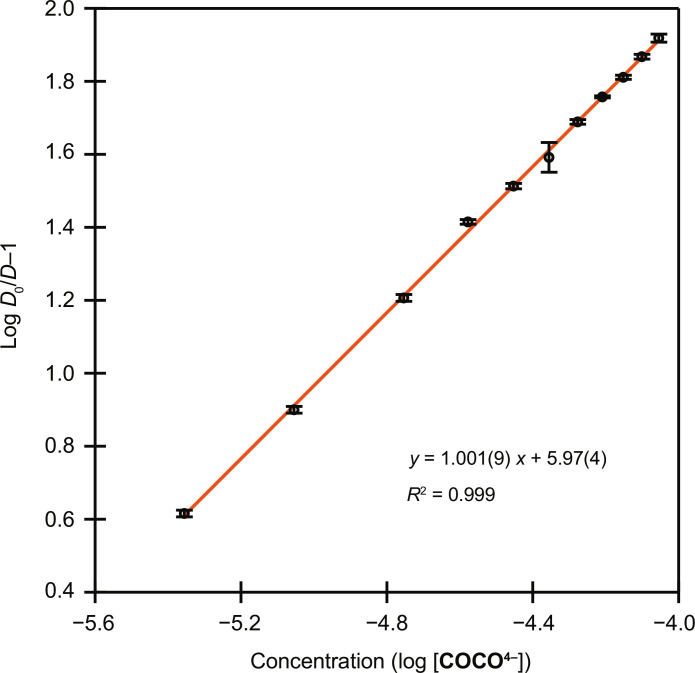
Determination of Ra(COCO)^2−^_(*aq*)_ stoichiometry. The logarithm of the ratio of distribution ratios of Ra^2+^ when complexed by **COCO**^**4−**^_**(*aq*)**_ (*D*) versus uncomplexed (*D*_0_) plotted as a function of log **COCO**^**4−**^_**(*aq*)**_ concentration (pH 6.29 with ionic strength set to 0.2 M by NaCl). Slope analysis of the linear relationship between log *D*_0_/*D*–1 versus log [**COCO**^**4−**^_**(*aq*)**_] gave a ligand-to-metal stoichiometry of 1.0. The data represent the average of three replicates and data point uncertainty was determined as the SD from the mean at 1σ.

A metal-ligand stoichiometry of 1:1 for complexation of ^223^Ra by **COCO**^**4−**^_**(*aq*)**_ was determined from the unity slope obtained by linear regression analysis of log(*D*_0_/*D* − 1) versus log[**COCO**^**4−**^_**(*aq*)**_] (Fig. 4). Repeating the cation exchange experiments at other pH values in the range of 5.92 to 7.68 produced data points overlapping with those from the original study at pH 6.29. These results supported the absence of protonated complexes in solution over the investigated pH range. Therefore, β_app_ can be taken as the pH-independent stability constant, or *K*_ML_ value, of the **Ra(COCO)**^**2−**^_**(*aq*)**_ complex. The magnitude of this stability constant was lower in comparison to those measured under identical experimental conditions for the Ra^2+^ complexes of 2,2′,2″,2‴-(1,4,7,10-tetraazacyclododecane-1,4,7,10-tetrayl)tetraacetic acid (DOTA) (log *K*_ML_ = 7.82) and macropa (*K*_ML_ = 10.00), two leading macrocyclic chelating agents for nuclear medicine applications (*7*). However, it is worth noting that at physiological pH 7.4, **COCO**^**4−**^_**(*aq*)**_ retains all its affinity for Ra^2+^ (log *K*′_ML_ = 5.97) by virtue of its low chelator basicity, which is reflected by complete deprotonation of **COCO**^**4−**^_**(*aq*)**_ at this pH. By contrast, the stabilities of the Ra(DOTA)^2−^ and Ra(macropa) complexes at pH 7.4 are reduced to log *K*′_ML_ = 4.29 for DOTA [lower than **COCO**^**4−**^_**(*aq*)**_] and 9.28 for macropa [higher than **COCO**^**4−**^_**(*aq*)**_]. Lowered stability for DOTA and macropa at this pH results from high ligand basicities, which essentially leads to competition between Ra^2+^ and H^1+^ for binding to DOTA and macropa. Collectively, these results demonstrated that **COCO**^**4−**^_**(*aq*)**_ had reasonably high affinity for Ra^2+^ near neutral pH.

### Characterization of Ba(H_2_COCO) and Sr(H_2_COCO)

**Fig. 5.**
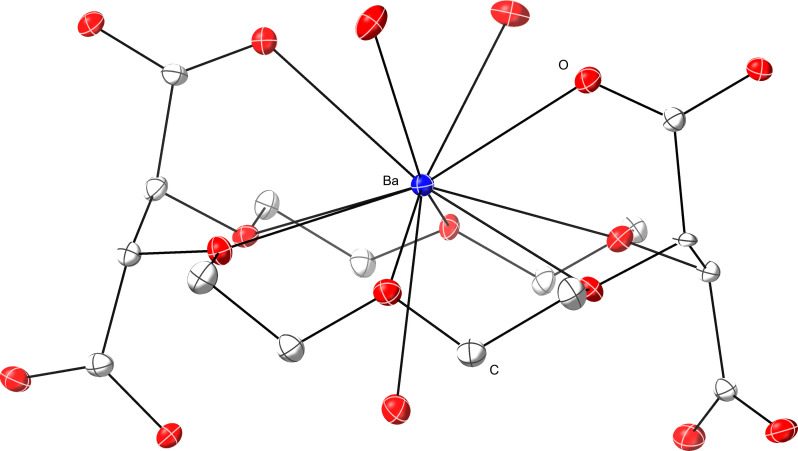
Representation of the single-crystal XRD data for Ba(H_2_COCO). Only one of two independent molecules in the asymmetric unit is shown at the 50% thermal ellipsoid probability. Hydrogens and cocrystallized water have been omitted. Ba = blue, O = red, C = gray.

**Fig. 6.**
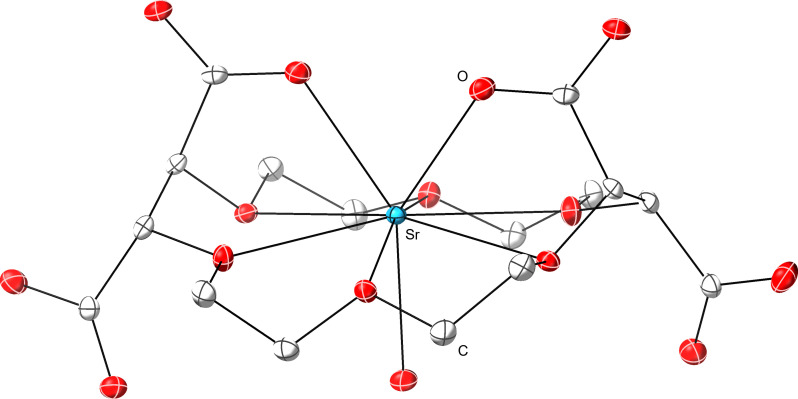
Representation of the single-crystal XRD data for Sr(H_2_COCO). Ellipsoids are shown at the 50% probability level. Hydrogens and cocrystallized solvent have been omitted. Sr = cyan, O = red, C = gray.

Complexation chemistry between **H**_**4**_**COCO** and nonradioactive isotopes of Ba^2+^ and Sr^2+^ was studied to bolster the notion that **H**_**4**_**COCO** was well suited for heavy alkaline earth complexation (*42*–*44*) and to extrapolate results to Ra^2+^_(*aq*)_ from its lighter congeners. Thus, **H**_**4**_**COCO** was mixed with barium(II) nitrate [Ba(NO_3_)_2_] or strontium(II) nitrate [Sr(NO_3_)_2_] in a solution of H_2_O and ethanol (EtOH). Colorless rectangular crystals were grown by slow evaporation of the solution over several days. Characterization of these crystals by single-crystal XRD revealed that the neutral, di-protonated complex **M(H**_**2**_**COCO)** (M = Ba^2+^, Sr^2+^) formed in high yield (80 to 90%) from aqueous/ethanolic solutions (no added external base; Fig. 1). The solid-state structure of **Ba(H**_**2**_**COCO)** (Fig. 5) was similar to that of **Sr(H**_**2**_**COCO)** (Fig. 6). For example, in both molecules, the Ba^2+^ and Sr^2+^ cations were bound through single oxygen atoms (i.e., κ^1^ coordination) associated with two of the pendant carboxylates on the 18-crown-6 periphery. There were also six M–O_crown_ interactions with ethereal oxygen atoms from the macrocyclic backbone. The Ba–O_crown_ distances ranged from 2.841(3) to 2.974(3) Å (mean = 2.90 ± 0.04 Å). The smaller Sr^2+^ ion formed Sr–O_crown_ bonds [2.684(3) and 2.797(3) Å; mean = 2.73 ± 0.05 Å] that were about 0.17 Å shorter than those observed in **Ba(H**_**2**_**COCO)**, as expected based on the 0.26 Å difference in Ba^2+^ (11 coordinate) and Sr^2+^ (9 coordinate) ionic radii (*38*). Note that all uncertainties for averaged bond distances have been reported as standard devations (SDs) from the mean at 1σ.

The solid-state structures of **M(H**_**2**_**COCO)** indicated that **H**_**2**_**COCO**^**2−**^ was insufficiently large to sterically saturate the Ba^2+^ and Sr^2+^ coordination spheres. In the **Ba(H**_**2**_**COCO)** case, for which the asymmetric unit contained two molecules, the Ba^2+^ dication was not completely contained within the crown ether ring of the **H**_**2**_**COCO**^**2−**^ complexing agent. Instead, it sat 0.912(5) Å above the least squares plane defined by ethereal oxygen atoms. This arrangement enabled two carboxylate groups to bind Ba^2+^ and positioned the other two carboxylic acid groups pointed away from and unable to interact with Ba^2+^. The Ba–O_carboxylate_ bond distances (mean 2.857 ± 0.016 Å) were similar to the Ba–O_crown_ distances discussed above. Additional evidence for steric unsaturation came from **Ba(H**_**2**_**COCO)** having three barium-bound H_2_O ligands; the dehydrated compound did not crystallize. Two aquo ligands were on the same face of the crown ether ring as Ba^2+^ (mean distance = 2.813 ± 0.013 Å). Meanwhile, the third aquo ligand reached through the crown ether ring from the face opposite barium (Ba–O_H2O_ distance = 2.927 ± 0.008 Å). Through-ring binding of water in related crown ether complexes has been observed previously (*54*–*57*). The through-ring Ba–O_H2O_ distance in **Ba(H**_**2**_**COCO)** was approximately 0.1 Å longer than that observed for the two *syn*-aquo ligands. The large coordination number of 11—two carboxylates, six ether functionalities, and three water ligands—highlighted the challenges associated with sterically saturating the large Ba^2+^ dication (ionic radius = 1.57 Å for a coordination number of 11) (*38*).

Many aspects of the **Sr(H**_**2**_**COCO)** solid-state structure were similar to **Ba(H**_**2**_**COCO)**. First and foremost, data from **Sr(H**_**2**_**COCO)** suggested that **H**_**2**_**COCO**^**2−**^ was incapable of sterically saturating the large Sr^2+^ dication. Notably, **H**_**2**_**COCO**^**2−**^ did not completely envelop the Sr^2+^ dication into the oxygen-containing ethereal plane, and Sr^2+^ needed additional H_2_O to fill out its coordination sphere. This Sr-bound aquo ligand reached through the crown ether ring to interact with Sr^2+^ (Sr–O_H2O_ distance = 2.505 ± 0.003 Å). Alongside these similarities were notable differences between the Sr^2+^ and Ba^2+^ structures. The 0.349(3)-Å displacement of Sr^2+^ from the ethereal oxygen plane was 0.6 Å less than that observed for Ba^2+^, and only one H_2_O ligand was bound to Sr^2+^ in **Sr(H**_**2**_**COCO)** versus three in **Ba(H**_**2**_**COCO)**. Hence, the overall coordination number for Sr^2+^ in **Sr(H**_**2**_**COCO)** was only 9 compared to 11 for Ba^2+^. All of these structural changes can be rationalized by the differences in ionic radii for the larger Ba^2+^ (1.57 Å) versus smaller Sr^2+^ (1.31 Å) dications (*38*). Extrapolating to the even bigger Ra^2+^ dication (1.7 Å; 12 coordinate) led us to predict that **RaCOCO**^**2−**^_**(*aq*)**_ would take on three, if not more, water molecules in its inner coordination sphere, giving a coordination number ≥ 11. We also reasonably speculated that Ra^2+^ might be held further from the ethereal oxygen plane than that observed for Ba^2+^ and Sr^2+^.

**Fig. 7.**
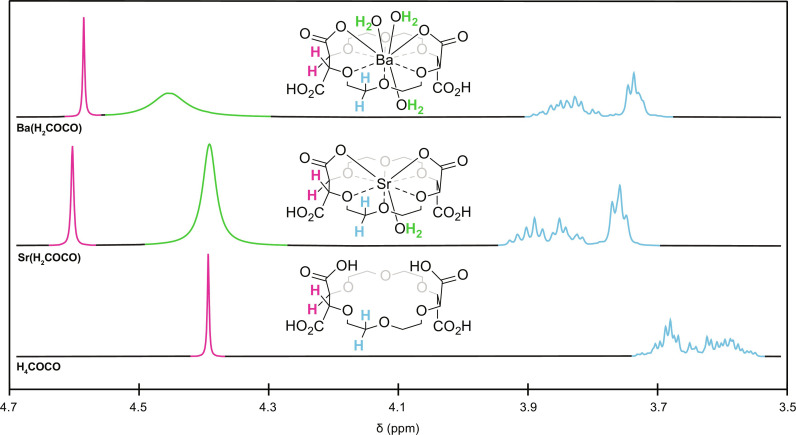
^1^H NMR spectra of Sr(H_2_COCO), Ba(H_2_COCO), and free H_4_COCO (in CD_3_CN). Pink, green, and blue peaks were assigned on the basis of the inserted structures.

Although crystals of **Ba(H**_**2**_**COCO)** and **Sr(H**_**2**_**COCO)** would not redissolve after they formed, we obtained NMR spectroscopic data that suggested the **M(H**_**2**_**COCO)** (M = Ba, Sr) solid-state structure reasonably described **M(H**_**2**_**COCO)** speciation when dissolved in organic solvents. That is, treating **H**_**4**_**COCO** with 1 equivalent of barium(II) triflate [Ba(OTf)_2_] or strontium(II) triflate [Sr(OTf)_2_] in CD_3_CN shifted the crown ether resonances downfield of the parent **H**_**4**_**COCO** ligand by approximately 0.2 parts per million (ppm) (Fig. 7). This downfield shift arose from deshielding of crown ether protons by the (weakly) electron withdrawing Ba^2+^ and Sr^2+^ cations. This peak shift was consistent with chelation via M–O_carboxylate_ and M–O_crown_ linkages, as observed in the solid-state structures. Each ^1^H NMR spectrum displayed a set of multiplets (–C*H*_2_–; δ 3.92 to 3.67 ppm, Ba^2+^; δ 3.95 to 3.71 ppm, Sr^2+^) and a sharp singlet [–C*H*(CO_2_H)–; δ 4.60 ppm, Ba^2+^; δ 4.62 ppm, Sr^2+^] in addition to a broad resonance attributable to metal-bound (adventitious) water and HOTf.

Additional support for our conclusion that **M(H**_**2**_**COCO)** existed in organic solution came from two series of NMR experiments. In the first series, we measured diffusion coefficients via diffusion ordered spectroscopy (DOSY). Those data showed that **Ba(H**_**2**_**COCO)** [diffusion coefficient = 7.31(5) × 10^−10^ m^2^/s] and **Sr(H**_**2**_**COCO)** [diffusion coefficient = 7.44(2) × 10^−10^ m^2^/s] moved slower through solution than the free ligand [diffusion coefficient = 9.38(5) × 10^−10^ m^2^/s]. We rationalized these results as indicating that **Ba(H**_**2**_**COCO)**_**(*CD3CN*)**_ and **Sr(H**_**2**_**COCO)**_**(*CD3CN*)**_ had higher molecular weights than **H**_**4**_**COCO**_**(*CD3CN*)**_, as expected if Ba^2+^_(*CD3CN*)_ and Sr^2+^_(*CD3CN*)_ were chelated by **H**_**2**_**COCO**^**2−**^_**(*CD3CN*)**_ in solution. The second series of experiments involved mixing the **Ba(H**_**2**_**COCO)** and **Sr(H**_**2**_**COCO)** samples together in the presence of excess ligand [i.e., 1:1:1 molar ratio of **Ba(H**_**2**_**COCO)**:**Sr(H**_**2**_**COCO)**:**H**_**4**_**COCO**] in CD_3_CN. After equilibrating the three-component solution for 2 days, we observed three well-resolved methine resonances associated with each of **Ba(H**_**2**_**COCO)**, **Sr(H**_**2**_**COCO)**, and **H**_**4**_**COCO** (see the Supplementary Materials). Analogous results were obtained by ^13^C NMR spectroscopy. These data demonstrated that resonances corresponding to **Ba(H**_**2**_**COCO)**, **Sr(H**_**2**_**COCO)**, and **H**_**4**_**COCO** were discernible by ^1^H and ^13^C NMR spectroscopy and that Ba^2+^ and Sr^2+^ were held tightly within the **H**_**2**_**COCO**^**2−**^ binding pocket.

**Fig. 8.**
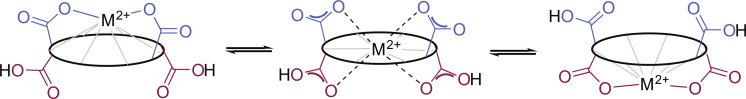
Depiction of proposed exchange process. A proposed fluxional process for explaining the symmetry of the observed ^1^H NMR spectra (see Fig. 7). This process could be accessible for **M(H**_**2**_**COCO)** (M = Ba^2+^, Sr^2+^) as described below.

One unexpected aspect from the **M(H**_**2**_**COCO)** ^1^H NMR data was equivalency for all four ligand methine resonances. Although similar observations have been made previously in related crown ether complexes of Ba^2+^, Sr^2+^, Ca^2+^, and K^+^, the origin of methine equivalency is often difficult to explain (*44*). The splitting pattern in our ^1^H NMR data suggested that **M(H**_**2**_**COCO)** had higher molecular symmetry in solution than would be expected for a complex that contained an out-of-plane metal, as observed by XRD (vide supra). That is, one would naïvely expect two inequivalent signals corresponding to diastereotopic methine protons. Consequently, it seemed likely that a dynamic process was occurring faster than the time scale of the NMR experiment. We posited that M^2+^ slipped from one face of the crown ether to the other rapidly in solution, as shown in Fig. 8. This shift would require M–O_carboxylate_ bond breaking, movement of M^2+^ through the crown ether plane, and M^2+^–O_carboxylate_ bond formation with backside carboxylate functionality (Fig. 8). Molecular motion through the crown ether would also likely necessitate loss of one bound aquo ligand as the M^2+^ cation passed through the crown ether binding pocket and then regaining of a new aquo ligand upon emerging on the other side. Concomitantly, the two protons associated with free carboxylate functionalities would have contrary molecular motion to M^2+^ and essentially swap from being bound by one set of carboxylates to the other.

**Fig. 9.**
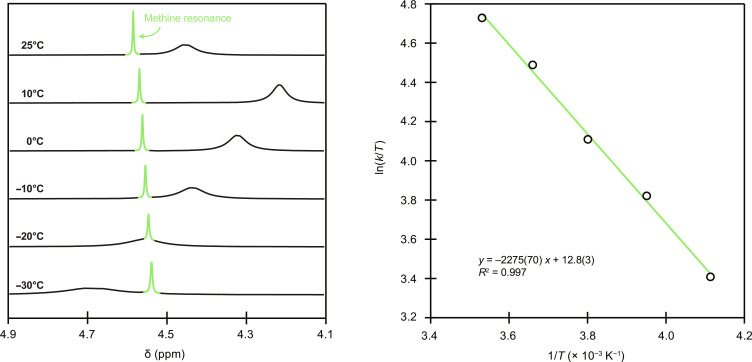
Variable temperature NMR experiments for Ba(H_2_COCO) in CD_3_CN. (**Left**) Variable temperature ^1^H NMR spectra of **Ba(H**_**2**_**COCO)**. (**Right**) Experimental fit to the Eyring equation. Errors are reported at 1σ.

Attempts to better characterize fluxional processes accessible to **Ba(H**_**2**_**COCO)** using variable temperature techniques were challenging. For instance, the most informative data came from CD_3_CN solutions, but peak coalescence was not observed above the CD_3_CN solvent freezing point (−46°C). We overcame this obstacle by collecting ^1^H NMR spectra at various temperatures (from −30° to +25°C; Fig. 9, left) and conducting line-width analysis (*58*) on the methine resonance. This enabled us to calculate chemical exchange rate constants *k_i_* at variable temperatures via the following equation

$$k_{i}=\frac{1}{2}\pi {\left(∆\nu_{0}\right)}^{2}{\left[{\text{FWHM}}_{i}-{\text{FWHM}}_{0}\right]}^{-1}$$ (3)

where Δν_0_ was the peak separation below the coalescence temperature, FWHM*_i_* was the full width at half maximum of the methine resonance broadened by exchange effects at variable temperature *i*, and FWHM_0_ was the full width at half maximum of the methine resonance at 25°C. This analysis relied on the assumption that peak separation below coalescence—which we were unable to observe—was equivalent to the typical value of 0.17 ppm (*58*) or 67 Hz on our 400-MHz spectrometer. The experimentally determined rate constants *k_i_* were fit to a linearized form of the Eyring equation, and the following kinetic parameters for the dynamic process were obtained: Δ*H*^‡^ = 4.5(1) kcal/mol; ΔS^‡^ = −0.0218(5) kcal/mol•K; Δ*G*_298K_^‡^ = 11.0(1) kcal/mol (Fig. 9, right); errors are reported as one SD from the mean. Those results were consistent with rapid exchange for the fluxional process depicted in Fig. 8. The free-energy barrier [11.0(1) kcal/mol] was also in excellent agreement with DFT model that we developed (vide infra) and suggested that side-to-side movement of Ba^2+^ through the crown ether would be easily achieved at room temperature.

### DFT analysis of *Ba(H_2_COCO)* fluxionality

**Fig. 10.**
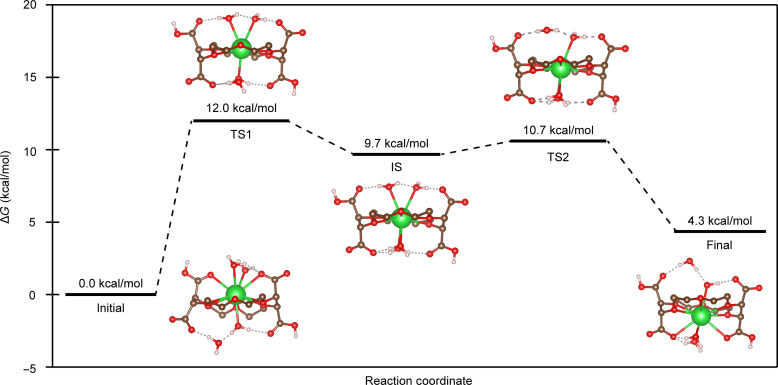
DFT reaction coordinate for Ba(H_2_COCO) fluxionality. The reaction path and free energy (Δ*G*, kcal/mol; 298.15 K) profile for the movement of Ba^2+^ through the **H**_**2**_**COCO**^**2−**^ crown ether ring are shown. Calculated structures for the initial state, transition state 1 (TS1), intermediate state (IS), transition state 2 (TS2), and final state geometries are included. In these representations, the hydrogen atoms on the crown ether were omitted. Δ*H*^‡^ for initial → TS1 = 9.6 kcal/mol; Δ*H* for TS1 → IS = −0.2 kcal/mol; Δ*H*^‡^ for IS → TS2 = 0.1 kcal/mol; Δ*H* for TS2 → final = −5.7 kcal/mol.

To rationalize the unexpectedly high symmetry of the solution-phase ^1^H NMR data, we used computational methods to interrogate the free-energy surface corresponding to movement of Ba^2+^ through the **H**_**2**_**COCO**^**2−**^ crown ether ring (Fig. 10). This effort combined DFT and nudged elastic band (NEB) calculations (*59*). To begin, an initial geometric conformation was identified on the basis of the single-crystal XRD data for **Ba(H**_**2**_**COCO)**. To simplify the calculation space, we ignored energetic contributions associated with movement of carboxylate protons from one side of the crown ether plane to the other by placing these protons on carboxylate functional groups that were on opposite sides of the crown ether ligand. This arrangement also produced the lowest total energy of all configurations sampled (see the Supplementary Materials).

Geometric configurations—and corresponding energetics—were obtained, and the molecular motion coordinate diagram shown in Fig. 10 was generated. To start, we identified a reasonable intermediate state (IS) configuration. The geometry for this **Ba(H**_**2**_**COCO)** IS configuration was optimized such that the Ba^2+^ cation was contained (approximately) within the crown ether plane. There were also four H_2_O ligands in the Ba^2+^ binding pocket vicinity, two on each side of the crown ether complexing agent. Three of these H_2_O ligands formed bonds with Ba^2+^, while the fourth H_2_O ligand bridged a Ba-bound H_2_O ligand to a carboxylate functional group through hydrogen bonding interactions. In this IS configuration, there were no close Ba–O_carboxylate_ interactions. Breaking one of the four Ba−O_H2O_ bonds forced Ba^2+^ to slip to the opposite side of the crown ether, initiated Ba−O_carboxylate_ bond formation, and created new hydrogen bonding interactions between free H_2_O, Ba-bound H_2_O, and a free carboxylate functional group. Geometries associated with all three of these configurations corresponded to local minima on the free-energy surface in the reaction path in Fig. 10. The final state closely resembled the initial state, but it accounted for possible unfavorable buckling of the macrocycle backbone. The structures with Ba^2+^ bound out of the crown ether plane (i.e., initial and final) were lower in energy than geometric configurations where Ba^2+^ was held within the crown ether plane, in agreement with the experimental structure of **Ba(H**_**2**_**COCO)** determined by single-crystal XRD.

Energy barriers and transition states for Ba^2+^ migration between the local minima were obtained with climbing image NEB calculations. Further refinements of transition state configurations and energies were obtained with a transition state search starting from maxima obtained via NEB. This step was necessary to mitigate the possibility of missing a true maximum, owing to the small number of images in such a long reaction path. Note that the model described in Fig. 10 neglected surrounding solvent molecules. In reality, these ignored “spectators” could facilitate hydrogen bond formation—or other electrostatic interactions with **Ba(H**_**2**_**COCO)**—and further decrease energy barriers for requisite geometric changes. Accounting more comprehensively for the outer sphere interactions should facilitate movement of Ba^2+^ from side to side of the **H**_**2**_**COCO**^**2−**^ ligand. Hence, we regarded the energy barriers reported here as likely upper energetic bounds.

The free-energy surface in Fig. 10 provided a model for Ba^2+^ side-to-side movement through the **H**_**2**_**COCO**^**2−**^ crown ether ring. This geometric change required Ba^2+^ to take on a fourth H_2_O ligand through a transition state (TS1), which had the highest free-energy barrier (Δ*G*^‡^ = 12.0 kcal/mol) on the energy surface sampled. The magnitude for this rate-limiting activation energy was quite similar to that determined experimentally (11.0 ± 0.1 kcal/mol) by NMR spectroscopy measurements. This free-energy barrier was not large and could easily be overcome by absorbing thermal energy from the surroundings at room temperature. The small changes in free energy required for subsequent cascade from TS1 → IS (−2.3 kcal/mol), IS → TS2 (+1.0 kcal/mol), and TS2 → Final (−6.4 kcal/mol) suggested that the Ba−O_H2O_ bonds were weak and labile. Given that (i) movement of Ba^2+^ through the crown ether appeared to be reliant on Ba−O_H2O_ bond formation/breakage and (ii) the energies associated with Ba−O_H2O_ bond forming and breaking reactions were small in magnitude, we concluded that Ba^2+^ movement through the crown ether ring reasonably explained the methine equivalency in the ^1^H NMR spectrum of **Ba(H**_**2**_**COCO)**. We also inferred that a similar reaction pathway accounted for the symmetry in the ^1^H NMR spectrum of **Sr(H**_**2**_**COCO)**.

## DISCUSSION

Here, some aspects of heavy alkaline earth chelation chemistry have been described. We found that the crown ether derivative **H**_**4**_**COCO** binds Ra^2+^, Ba^2+^, and Sr^2+^ dications. The single-crystal XRD data suggested that the **H**_**2**_**COCO**^**2−**^ ligand was insufficiently large to fill the M^2+^ coordination sphere. Instead, a combination of **H**_**2**_**COCO**^**2−**^ and several aquo ligands were required to achieve steric saturation. For Sr^2+^ and Ba^2+^, stability was demonstrated by characterizing the **M(H**_**2**_**COCO)** complexes using standard methods (e.g., NMR and XRD). For Ra^2+^, it was not possible to characterize complexation by **H**_**4**_**COCO** in our laboratory using analogous techniques because radium is scarce. Instead, we harvested the short-lived ^223^Ra radionuclide from an ^227^Ac source, purified the obtained ^223^Ra from other ^227^Ac progeny products, and prepared a **Ra(COCO)**^**2−**^_**(*aq*)**_ coordination complex in 30% yield. The anionic **Ra(COCO)**^**2−**^_**(*aq*)**_ coordination complex exhibited a reasonably high stability constant [log *K*_**Ra(COCO)2–**_ = 5.97 ± 0.01] in MES-buffered solutions. All three of the **M(H**_***x***_**COCO)**^***x*–2**^ coordination complexes were stable under air and at neutral pH.

The ^1^H NMR data from **M(H**_**2**_**COCO)** (M = Sr, Ba) and the calculations carried out on **Ba(H**_**2**_**COCO)** provided insight into the solution-phase dynamics accessible to M^2+^ cations within the **M(H**_**2**_**COCO)** framework. One key finding was that migration of M^2+^ from side to side of the crown ether was mediated by M–O_H2O_ bond breaking and forming reactions. DFT calculations indicated that movement of the M^2+^ cation through the **H**_**2**_**COCO**^**2−**^ ring could reasonably account for methine equivalency in the ^1^H NMR spectra. Notably, the free-energy barrier facing side-to-side movement of Ba^2+^ through the **H**_**2**_**COCO**^**2−**^ crown ether cavity was calculated by DFT to be 12 kcal/mol. This theoretical value compared exceptionally well with the experimentally determined value of 11.0(1) kcal/mol and showed that this energic barrier could easily be overcome from thermal energy at room temperature. In our model, numerous variables contributed to stability of out-of-plane bound M^2+^. The most notable contributors were hydrogen bonding interactions among adjacent H_2_O molecules, hydrogen bonding between aquo ligands and carboxylate functional groups, and destabilizing buckling of the crown ether that occurred when M^2+^ sat outside of the crown ether ring plane.

Complexation of Ra^2+^, Ba^2+^, and Sr^2+^ by **H**_**4**_**COCO** was intriguing on other levels. First and foremost, these data boasted a robust method to chelate Ra^2+^_(*aq*)_ and form a rare example of a radium coordination complex, namely, **Ra(COCO)**^**2−**^_**(*aq*)**_. The present results also added to a growing body of knowledge for heavy alkaline earth coordination chemistry, reinforced the potential for crown ethers as ligands for Ra^2+^, Ba^2+^, and Sr^2+^ (*42*–*44*), and provided insight into ways that more stable chelates could be designed. Our data demonstrated that multidentate and/or large chelators are needed to meet the coordination number requirements for steric saturation of heavy alkaline earth elements: 9 for Sr^2+^ in **Sr(H**_**2**_**COCO)**, 11 for Ba^2+^ in **Ba(H**_**2**_**COCO)**, and likely greater than 11 for Ra^2+^ in **Ra(COCO)**^**2−**^_**(*aq*)**_. Our results also suggested that chelators with large negative charges facilitate binding heavy alkaline earth metals, whose weakly polarizing properties impede their ability to attract ligands and to form stable coordination complexes with neutral complexation agents. We infer that larger binding constants—and perhaps even selective alkaline earth complexation—could be achieved if the chelator’s anionic charge and number of donor sites on the macrocyclic backbone were tailored to these M^2+^ cations.

We are excited by these results and their implications for use of anionic cyclic polyethers as chelators for Ra^2+^, Ba^2+^, and Sr^2+^. It is our hope that insight from these studies triggers creative ideas that help researchers overcome contemporary struggles with M^2+^ chelation chemistry. From this perspective, data reported here provide a “proof of principle” that may inspire others to explore heavy alkaline earth coordination chemistry—especially in terms of advancing use of Ra^2+^, Ba^2+^, and Sr^2+^ in applied medical technologies, as catalysts that enable organic chemical transformations, and even within the field of small-molecule binding and activation.

## MATERIALS AND METHODS

### General methods

*Caution! ^227^Th, ^227^Ac, ^223^Ra, and their radioactive decay products are highly radioactive* α*-,* β*-, and* γ*-emitting radionuclides.* Hence, all studies with these isotopes were conducted in laboratories equipped with continuous air monitors; HEPA-filtered fume hoods (certified), continuous air monitors; and monitoring equipment appropriate for α-, β-, and γ-particle detection. Entrance to the laboratory space was controlled with a hand and foot monitoring instrument for α-, β-, and γ-emitting isotopes and a full-body personnel contamination monitoring station. All reactions were performed in open face fume hoods with no effort to exclude air or ambient moisture. The ^227^Ac radionuclide was obtained from the National Isotope Production Program. The ^223^Ra radioisotope was purified, as described below, before use in Chelex 100 experiments. The ^227^Ac radioisotope was dissolved in HNO_3(*aq*)_ (8 M) before use and was used as a source for harvesting the ^227^Th and ^223^Ra progenies (vide infra).

For ^223^Ra stability constant measurements, a slightly different procedure was used: ^223^Ra was purchased from the National Isotope Development Center as a nitrate salt with a specific activity of 1.896 × 10^15^ Bq/g or 5.123 × 10^4^ Ci/g (carrier free), a radionuclidic purity of 99.99%, and a chemical purity of 99%. Upon receipt, it was reconstituted in 10 mM HCl (Fisher Optima HCl and Fisher ultra-trace elemental analysis grade H_2_O). The radionuclidic purity and activity of ^223^Ra was verified by high purity germanium (HPGe) γ spectrometry (Gamma Analyst Integrated Gamma Spectrometer, Canberra). The detector energy and efficiency were calibrated using a mixed γ point source containing ^57^Co, ^60^Co, ^88^Y, ^109^Cd, ^113^Sn, ^137^Cs, ^139^Ce, ^203^Hg, and ^241^Am, traceable to the National Institute of Standards and Technology (NIST) and supplied by Eckert & Ziegler Analytics (Atlanta, GA, USA). Counting dead time was maintained below 5%. Spectra were analyzed using Genie 2000 software (v3.2.1, Canberra). Working solutions were subsequently prepared at 560 to 740 Bq ^223^Ra/μl (0.015 to 0.02 μCi ^223^Ra/μl) before each experiment by further dilution with 10 mM HCl.

Dowex 50W X8 resin (hydrogen form, 200 to 400 mesh) was purchased from Sigma-Aldrich. The resin was converted to the Na^1+^ form as described previously (*7*). Buffers for cation exchange experiments were prepared using ultra-trace elemental analysis grade H_2_O (Fisher Chemical), Suprapur NaCl (99.99%; Sigma-Aldrich), and MES hydrate (≥99.5%; BioXtra, Sigma-Aldrich) or Hepes (≥99.5%; Sigma-Aldrich). Each buffer was adjusted to the desired pH using a small volume of concentrated sodium hydroxide (semiconductor grade, 99.99% trace metals basis; Sigma-Aldrich) in ultra-trace elemental grade H_2_O. Acetonitrile (MeCN, anhydrous; Thermo Fisher Scientific), ethanol (EtOH, anhydrous; Sigma-Aldrich), deuterated water (D_2_O; Cambridge Isotopes), deuterated acetonitrile (CD_3_CN, 99.8%; Sigma-Aldrich), Ba(NO_3_)_2_, Ba(OTf)_2_, Sr(NO_3_)_2_, and **H**_**4**_**COCO** acid (Sigma-Aldrich) were obtained commercially and used without further purification. Strontium triflate was synthesized by the action of triflic acid (HOTf, 99.9%; Sigma-Aldrich) on strontium carbonate, which was precipitated from aqueous strontium chloride (99.9%; Sigma-Aldrich) by sodium carbonate. Aqueous nitric acid [HNO_3(*aq*)_, Optima Grade, Thermo Fisher Scientific] and aqueous hydrochloric acid [HCl_(*aq*)_, Optima Grade, Thermo Fisher Scientific] were obtained commercially and used as received. For the ^223^Ra/Chelex 100 experiments, water was deionized and passed through a Barnstead water purification system until a resistivity of 18 megohm•cm was achieved; this water (18 megohm•cm) was further purified by distillation using a Teflon distillation apparatus. DGA resin, branched (4 μm; Eichrom), pre-filter (100 to 150 μm; Eichrom), AG 1-X8 (45 to 106 μm; Bio-Rad), and Chelex 100 (100 to 350 μm; Bio-Rad) resins were purchased commercially and used as received. The 50-ml conical tubes used in this study were made of polypropylene.

### NMR spectroscopy

All NMR experiments were conducted using a Bruker Avance III 400 MHz NMR and processed with MestReNova software. ^1^H NMR chemical shifts are given in parts per million with respect to solvent residual peak (CD_3_CN, δ 1.94). ^13^C NMR chemical shifts are given in parts per million with respect to solvent peaks (CD_3_CN δ 118.3, 1.3). Multiplicities are described as s = singlet, m = multiplet, and br s = broad singlet.

### High-resolution mass spectrometry

HRMS analysis was performed at the University of Texas at Austin Mass Spectrometry Facility. Data were collected in positive mode with electrospray ionization (ESI) with an Agilent 6530 Accurate Mass Q-TOF LC/MS.

### Crystallography

Single crystals of **Ba(H**_**2**_**COCO)** and **Sr(H**_**2**_**COCO)** were coated in Paratone-N oil (Hampton Research), mounted on Mitegen Cryoloops, and placed on a Bruker D8 Quest diffractometer. The x-ray instrument contained a molybdenum x-ray tube (λ = 0.71073 Å). Data were worked up in Apex III software to determine unit cells and to control the data acquisition. The crystal structures were solved using SHELX (*60*, *61*) as implemented in OLEX2 software (*62*). All structural data in this manuscript were archived in the Cambridge Crystal Data Center as CIF files (CCDC deposition numbers 2237641 and 2237642).

### Preparation of a ^223^Ra^2+^ stock solution

A three-step purification process was used to harvest a radiochemically pure ^223^Ra stock solution (7.8 × 10^5^ Bq, 21 μCi) from an ^227^Ac solution (“generator solution”). The method reported here for isolating the ^223^Ra progeny from the ^227^Ac parent was similar to previously published procedures used to isolate pure stocks of other isotopes in the ^227^Ac decay chain (*46*, *47*).

#### Step 1: Removal of ^227^Th progeny

Under air and in an open front fume hood, a Bio-Rad column (10 ml) was loaded with an anion exchange resin (AG 1-X8, 5 ml, 200 to 400 mesh) suspended in Teflon-distilled H_2_O. The resin was covered with a plastic frit. The column was conditioned sequentially with H_2_O (3 × 5 ml), HCl_(*aq*)_ (0.1 M, 3 × 5 ml), H_2_O (3 × 5 ml), and lastly HNO_3(*aq*)_ (8 M, 3 × 5 ml). Next, a solution that contained the ^227^Ac^3+^_(*aq*)_, ^227^Th^4+^_(*aq*)_, and ^223^Ra^2+^_(*aq*)_ radionuclides was prepared for loading onto the column. It was crucial that the ^223^Ra-containing solution consisted solely of HNO_3(*aq*)_; otherwise, ^227^Th_(*aq*)_ would co-elute with ^227^Ac and ^223^Ra. Hence, before column loading, the generator solution was evaporated to a soft dryness using a hot plate under a rapidly flowing stream of filtered air. Note that we define the term “soft dryness” as the point at which all of the solution has evaporated; we use this term to remind the reader to avoid prolonged heating, which can bake the residue to the vessel and complicate subsequent dissolution. The resulting residue was dissolved in HNO_3(*aq*)_ (16 M). This process of evaporation and dissolution was repeated two more times to ensure a homogenous HNO_3(*aq*)_ (16 M) matrix. After evaporating the solution to a soft dryness for a third time, the residue was dissolved in HNO_3(*aq*)_ (8 M, 5 ml). This solution was loaded onto the anion column. Under these conditions, Th^4+^_(*aq*)_ was retained by the resin while Ra^2+^_(*aq*)_ and Ac^3+^_(*aq*)_ passed through the column with the load solution. The eluate containing Ra^2+^_(*aq*)_ and Ac^3+^_(*aq*)_ (column loading fraction) was collected into a conical tube (50 ml). Residual Ra^2+^_(*aq*)_ and Ac^3+^_(*aq*)_ retained by the resin were recovered in the same tube by washing the column with additional HNO_3(*aq*)_ (3 M, 3 × 5 ml). The solution was evaporated to dryness on a hot plate under a stream of filtered air. The yield for step 1 was 98% as determined by γ-spectrometry. The residue was carried forward to step 2, where ^227^Ac was separated from ^223^Ra progeny. Note that although the ^227^Th activity retained by this column was not used further in this study, it is still quite valuable. If desired, the ^227^Th activity can be recovered from this anion exchange column by washing the resin with HCl_(*aq*)_ (6 M, 3 × 5 ml) and eluting Th^4+^_(*aq*)_ with dilute HCl_(*aq*)_ (0.1 M, 3 × 5 ml).

#### Step 2: Isolation of ^223^Ra from ^227^Ac

Under air and in an open front fume hood, a Bio-Rad column (10 ml) was loaded with DGA resin and branched (1 ml, 50 to 100 μm; Eichrom) resin suspended in H_2_O. The resin was covered with a plastic frit and conditioned sequentially with H_2_O (3 × 3 ml), HCl_(*aq*)_ (0.1 M, 3 × 3 ml), H_2_O (3 × 3 ml), and HNO_3(*aq*)_ (6 M, 3 × 3 ml). The ^227^Ac and ^223^Ra eluate from step 1 was dissolved in HNO_3(*aq*)_ (6 M, 5 ml). This solution was loaded onto the column. Under these conditions, Ac^3+^_(*aq*)_ was retained by the resin and Ra^2+^_(*aq*)_ was passed through the column. The Ra^2+^_(*aq*)_ eluate was collected in a conical tube (50 ml). Residual ^223^Ra retained by the resin was eluted from the column by washing the resin with HNO_3(*aq*)_ (6 M, 3 × 1 ml). These wash fractions were collected in three separate conical tubes (50 ml), all of which were assayed using γ-spectrometry. All wash fractions that contained ^223^Ra were combined in an Erlenmeyer flask (250 ml), alongside the load fraction that contained the vast majority of the ^223^Ra activity. This solution was evaporated to a soft dryness using a hot plate under a rapidly flowing stream of filtered air. The resulting residue was dissolved in H_2_O (1 ml). The ^223^Ra activity was recovered in quantitative yield. The ^223^Ra residue was further purified in step 3. Note that although the ^227^Ac activity retained by the column was not used further in this study, it is still quite valuable because ^227^Ac decays to generate ^223^Ra. Hence, ^227^Ac was recovered from the resin by washing the column with dilute HCl_(*aq*)_ (0.1 M, 3 × 1 ml). These ^227^Ac fractions were collected into a single conical tube and used as a generator solution for subsequent ^223^Ra and ^227^Th harvesting activities by repeating steps 1 and 2.

#### Step 3: Purification with Pre-Filter Resin

Under air and in an open front fume hood, a Bio-Rad column (10 ml) was loaded with Pre-Filter Resin (3 ml, 100 to 150 μm). The resin was covered with a frit and the column conditioned with H_2_O (3 × 3 ml). The ^223^Ra solution from step 2—dissolved in H_2_O (1 ml)—was loaded onto the prefilter column. Under these conditions, ^223^Ra passed through the resin, and the column eluate was collected in a conical tube (50 ml). Residual ^223^Ra that remained on the resin was recovered by washing the column with more H_2_O (3 × 3 ml). This eluate was collected into the same conical tube that contained the ^223^Ra load solution. The solution (~10 ml) was evaporated to a soft dryness using a hot plate under a rapidly flowing stream of filtered air. Although no residue was visible upon removal of volatiles, the product was detected by the substantial γ-radiation emitted from the sample. This ^223^Ra residue was dissolved and quantitatively transferred into a conical tube (50 ml) using H_2_O (1.5 ml). Subsequent analysis by γ-spectrometry revealed that the stock solution was radiochemically pure and that ^223^Ra had been isolated in quantitative decay corrected yield (7.8 × 10^5^ Bq, 21 μCi ^223^Ra). This stock solution was used in the complexation studies with **H**_**4**_**COCO**.

### Complexation of ^223^Ra

#### Preparation of Ra(COCO)^2−^_(aq)_

Under air and in an open front fume hood, an aliquot (0.5 ml, 2.6 × 10^5^ Bq, 7.0 μCi ^223^Ra, 1.37 × 10^−7^ mg, 6.13 × 10^−10^ mmol, 3.69 × 10^11^ atoms) of the ^223^Ra stock solution was mixed with a ~ 370,000-fold excess of **H**_**4**_**COCO** (0.1 mg; 2.3 × 10^−4^ mmol) that had been dissolved in H_2_O (0.5 ml). The solution was set aside for 45 min at ambient temperature. Meanwhile, a Bio-Rad (3 ml) column loaded with Chelex 100 resin (2 ml; a mesh size of 100 to 200) was covered with a frit and conditioned with Teflon-distilled H_2_O (3 × 2 ml). Then, the solution containing ^223^Ra^2+^ and **H**_**4**_**COCO** was loaded onto the column. Under these conditions, the **Ra(COCO)**^**2−**^_**(*aq*)**_ coordination complex passed through the resin with the load solution. Free Ra^2+^—not complexed by **H**_**2**_**COCO**^**2−**^—was retained by the resin. The column was washed with H_2_O (3 × 1 ml). Analyses of the load and washing fractions using γ-spectrometry revealed that ^223^Ra (7.8 × 10^4^ Bq, 2.1 μCi, 30% yield) passed through the column.

#### Potentiometric titrations

The protonation constants of **H**_**4**_**COCO** were determined at 25°C and *I* = 0.2 M NaCl by potentiometric titration. Details of the titration setup can be found elsewhere (*7*). NaOH (0.2 M) was either purchased from Honeywell (Fluka, volumetric solution) or prepared using NaOH pellets (semiconductor grade, 99.99%; Sigma-Aldrich) and boiled water. The NaOH solution was standardized against potassium hydrogen phthalate (BioXtra, ≥99.95%; Sigma-Aldrich). HCl (0.1 M, Metrohm Certified Titrants) was titrated against tris base (Ultrapure Bioreagent, J.T. Baker) to verify its concentration. Before every titration, the electrode was calibrated in terms of the hydrogen-ion concentration by titrating a solution of standardized HCl (0.005 M) containing supporting electrolyte ([NaCl] = 0.195 M) with standardized NaOH. Data within the pH ranges of 2.3 to 3.2 and 10.8 to 11.3 were analyzed using the program GLEE (version 3.0.21) (*63*) to obtain the standard electrode potential (*E*_0_) and slope factor. The H_2_O ion product (p*K*_w_ = 13.74) was taken from the literature (*64*). A stock solution of **H**_**4**_**COCO** (~27 mM) was prepared in ultra-trace water. Its exact concentration was determined by quantitative ^1^H NMR spectroscopy using a known concentration of dimethyl sulfone as an internal standard (*n* = 3).

Protonation constants were measured by adding standardized NaOH to an aqueous solution (20 ml) of ligand (0.02 mmol), hydrochloric acid (0.1 mmol), and NaCl (3.9 mmol). The titration method used a drift limit of 0.1 mV min^−1^, a minimum wait time of 0 s, and a maximum wait time of 180 s between additions of aliquots of base. The data were refined using Hyperquad2013 (*65*). Only the proton concentration was admitted as a refinable parameter. The protonation constants, defined in Eq. 4, were calculated from the average of four independent titrations, with ~60 data points for each titration. These values are compiled in table S1. An overlay of titration curves can be found in fig. S1.

$$K_{\mathrm{ai}}=\frac{\left[H_{i}L\right]}{\left[H_{i-1}L\right]\left[H^{+}\right]}$$ (4)

#### ^223^Ra stability constant determination

The setup for these experiments has been described previously (*7*, *52*, *53*) but is reproduced here for completeness. MES (pH 5.92, 6.29, and 6.74) and Hepes (pH 7.68) buffers were prepared at a concentration of 0.025 M and a total ionic strength of 0.2 M (buffer + NaCl), and their pH was determined at 25°C using a glass electrode calibrated by titration of 0.005 M HCl/0.195 M NaCl from pH 2.3 to 11.3 with 0.2 M NaOH. A stock solution of **H**_**4**_**COCO** was prepared and quantified as described above. From this stock solution, a series of solutions of varying ligand concentration was prepared by further dilution with buffer. Specifically, preliminary scouting experiments were performed at pH 5.92, 6.74, and 7.68 with three different concentrations of **H**_**4**_**COCO** (10^−4^, 10^−5^, and 10^−6^ M, *n* = 1). Following these preliminary experiments, distribution experiments were performed in triplicate at pH 6.29 and 5 × 10^−6^ to 1 × 10^−4^ M **H**_**4**_**COCO** (11 data points per concentration series). Samples were prepared by adding aliquots (1 ml) of ligand solution to screw-capped polypropylene tubes containing 25 ± 0.5 mg of Dowex 50W X8 resin (hydrogen form, 200 to 400 mesh). Samples containing buffer only in the presence (*D*_0_) or absence (Ra_tot_) of resin were also prepared. All samples were spiked with 10 μl (560 to 740 Bq, 0.15 to 0.20 μCi) of ^223^Ra working solution and mixed by end-over-end rotation at 40 rpm at 25°C for 24 or 48 hours. Samples were then centrifuged at 8600 rpm for 3 min, and an aliquot (0.5 ml) of supernatant was added to 5 ml of Ultima Gold liquid scintillation cocktail. The liquid scintillation counter (LSC) samples were mixed by inversion and counted on a Tri-Carb 4910TR liquid scintillation counter (PerkinElmer). The energy window was set at 0 to 2000 keV. Samples were counted at least 15 hours after preparation to allow sufficient time for radioactive equilibrium to be reached between ^223^Ra and its decay chain. Counting of each sample was terminated once the 2σ uncertainty in the count rates reached 0.5% or after 1 hours, whichever criterion was reached first. Each sample count rate was decay corrected to the time at the start of the LSC analysis. The distribution coefficient (*D* value) of each sample was subsequently calculated as the ratio of activity in the resin versus activity in the aqueous phase (*A*_resin_/*A*_aq_) at equilibrium, wherein *A*_resin_ = *A*_total_ − *A*_aq_. No differences in the distribution data were observed between samples mixed for 24 hours versus 48 hours, indicating that the distribution measurements were taken at equilibrium.

From the distribution ratios of ^223^Ra in the absence (*D*_0_) or presence (*D*) of varying concentrations of **H**_**4**_**COCO**, a conditional cumulative stability constant, β_app_, of metal-ligand complexation can be determined according to Eq. 5 below

$$\frac{D_{0}}{D}-1=\beta_{\text{app}}\left[{\text{COCO}}^{4-}\right]$$ (5)

Fully deprotonated ligand concentration ([**COCO**^**4−**^]) in each sample was calculated from total ligand concentration, solution pH, and ligand protonation constants determined in 0.2 M NaCl. β_app_ is provided as the slope of the linear plot of *D*_0_/*D*–1 versus **COCO**^**4−**^ concentration. β_app_ is a conditional cumulative stability constant for metal-ligand complexation that is only valid for the pH at which it is determined. This overall constant is further defined in Eq. 6, assuming the formation of only 1:1 M:L complexes

$$\beta_{\text{app}}=\sum \beta_{\text{mhl}}{\left[H^{+}\right]}^{h}\left[L\right]$$ (6)

where β_mhl_ is the stability constant for the complex MH_h_L (*66*). A metal-ligand stoichiometry of 1:1 for complexation of ^223^Ra by **COCO**^**4−**^ was determined from the slope obtained through linear regression analyses of log (*D*_0_/*D*–1) versus log [**COCO**^**4−**^] (Fig. 4).

To derive the pH-independent stability constant, or log *K*_ML_ value, and the stepwise stability constants of any protonated metal-ligand complexes, preliminary scouting experiments were run at several different pH values (pH 5.92, 6.74, and 7.68, see above) (*67*). The data points from each pH were found to overlap to form a single line, supporting the absence of protonated complexes in solution over the pH range investigated (fig. S2). Therefore, β_app_ can be taken as the *K*_RaL_ value within this pH range. This finding is consistent with the results from protonation constant measurements of **H**_**4**_**COCO**, which reveal that **H**_**4**_**COCO** is nearly completely deprotonated by pH 5.92, the first pH value investigated in the series. Full distribution experiments were subsequently performed in triplicate at pH 6.29, and the log β_app_ (log *K*_RaL_) for **Ra(COCO)**^**2−**^_**(*aq*)**_ is reported as the average ± 1 SD (Fig. 3).

### Synthesis of Ba(H_2_COCO)

In an open front hood and with no attempt to exclude air and moisture, a scintillation vial (20 ml) was charged with Ba(NO_3_)_2_ (18 mg, 0.068 mmol). This white solid was dissolved in H_2_O (1 ml). Then, a solution of **H**_**4**_**COCO** (30 mg, 0.068 mmol, 1.0 equivalent) dissolved in ethanol (1 ml) was added. The resulting colorless solution was capped loosely and set aside in a fume hood. The next day, colorless crystals suitable for single-crystal XRD formed before the solution completely evaporated. The supernatant was discarded. Drying the crystals under high vacuum afforded the product as a white powder (35 mg, 89% with respect to the *des*-aquo complex). Because the crystalline solid was insoluble in common NMR solvents, NMR characterization was performed on the reaction mixture in situ: Ba(OTf)_2_ (8.7 mg, 0.020 mmol) and **H**_**4**_**COCO** (8.8 mg, 0.020 mmol, 1.0 equiv) were dissolved in CD_3_CN (0.6 ml). After about 5 min at room temperature, the mixture was analyzed by NMR spectroscopy and found to be analytically pure, except for adventitious H_2_O originating from the alkaline earth triflate or CD_3_CN.

^**1**^**H NMR** (400 MHz, CD_**3**_CN) δ 5.25 (br s), 4.60 (s, 4H), 3.92 to 3.80 (m, 8H), 3.80 to 3.67 (m, 8H). ^**13**^**C NMR** (101 MHz, CD_3_CN) δ 171.3, 80.1, 71.4, 71.2. **HRMS (ESI)** calculated for C_16_H_22_BaO_14_Na [M + Na − 3H_2_O]^1**+**^: 598.9956; found: 598.9956.

### Synthesis of Sr(H_2_COCO)

In an open front hood and with no attempt to exclude air and moisture, a scintillation vial (20 ml) was charged with Sr(NO_3_)_2_ (11 mg, 0.050 mmol). This white solid was dissolved in H_2_O (1 ml). Then, a solution of **H**_**4**_**COCO** (22 mg, 0.050 mmol, 1.0 equivalent) in ethanol (1 ml) was added. The resulting colorless solution was capped loosely and aged in a fume hood. The next day, colorless crystals suitable for single-crystal XRD formed before the solution completely evaporated. The supernatant was discarded. Drying the crystals under high vacuum afforded the product as a white powder (24 mg, 91% with respect to the *des*-aquo complex). Because the crystalline solid was insoluble in common NMR solvents, NMR characterization was performed on the reaction mixture in situ: Sr(OTf)_2_ (7.7 mg, 0.020 mmol) and **H**_**4**_**COCO** (8.8 mg, 0.020 mmol, 1.0 equivalent) were dissolved in CD_3_CN (0.6 ml). After about 5 min at room temperature, the mixture was analyzed by NMR spectroscopy and found to be analytically pure, except for adventitious water originating from the alkaline earth triflate or CD_3_CN. ^**1**^**H NMR** (400 MHz, CD_3_CN) δ 6.32 (br s), 4.62 (s, 4H), 3.95–3.83 (m, 8H), 3.83–3.71 (m, 8H). ^**13**^**C NMR** (101 MHz, CD_3_CN) δ 172.2, 79.2, 71.5, 70.7. **HRMS (ESI)** calculated for C_16_H_22_SrO_14_Na [M + Na − H_2_O]^1+^: 548.9959; found: 548.9962.

### Computational details

Kohn-Sham DFT (*68*, *69*) calculations were performed on the **Ba(H**_**2**_**COCO)•4H**_**2**_**O** molecule using the Amsterdam Density Functional (ADF 2022.103) package (*70*, *71*). To include the effect of the solvent into the electronic structure of the complex, we used the conductor-like screening model (COSMO) (*72*, *73*) as parameterized for water. Geometry optimizations were carried out by employing the generalized gradient approximation with Perdew-Burke-Ernzerhof (PBE) exchange-correlation functional (*74*). The Slater basis sets with the quality of triple-ζ plus two polarization functions (TZ2P) (*75*) and small frozen core approximation were used. The geometric structures of initial, intermediate, and final systems were fully optimized by DFT/PBE at the scalar-relativistic zero-order regular approximation (*76*) with gradient convergence of 10^−3^ Hartree/Å and energy convergence of 10^−5^ Hartree (see optimized structures in the Supplementary Materials). An approximation of the reaction path between initial, intermediate, and final systems was obtained by performing a linear interpolation as an initial guess. From this initial guess, the reaction path was obtained using the NEB methodology. The NEB reaction converges to a discretized version of the minimum energy path as defined by equally spaced points along the trajectory. Therefore, the actual transition state may be missed. A subsequent refinement of the transition state search was obtained using the climbing image algorithm, converging to a gradient of 10^−3^ Hartree/Å and energy convergence of 10^−5^ Hartree using the two highest energy intermediate images. The geometries of transition states (TS1 and TS2) were confirmed to be first-order saddle points by vibrational frequency calculations.

#### Starting configuration of the reaction

For a soft molecular complex, it is important to know the stability of its configurational space before exploring its energy profile along the reaction coordinates. We thoroughly investigated the energy landscape of the two protons (as indicated by blue spheres) on various carboxylic acid sites as shown in fig. S13. For these configurations in fig. S13A, the Ba^2+^ is on one side of crown ether, and the most stable configuration is C1 having the two protons on carboxylic acid substituents that are the opposite side of the ring with respect to the Ba^2+^. The configurations from C2 to C6 are higher in electronic energy by 0.79, 2.06, 2.70, 4.22, and 5.60 kcal/mol, respectively. Similarly, for these configurations in fig. S13B, the Ba^2+^ is in the other side of crown either, and the most stable configuration is C7. The configurations from C8 to C12 are higher in electronic energy.

#### DFT calculations of reaction mechanism

The relevant Ba–O bond lengths and total energies E optimized with DFT/PBE of initial system, intermediate state (IS), and final system are given in the Supplementary Materials. In the NEB calculation, we set 32 images and a rough approximation of the reaction path was built by performing a linear interpolation between the initial, intermediate, and final systems. The energy profile along the NEB minimum energy path is shown in fig. S14. During the NEB path optimization, a climbing image algorithm was used to drive the highest-energy image in the path to the transition state. There were two local highest-energy images as indicated by TS1* (tentative transition state 1) and TS2* (tentative transition state 2). A further refinement of the transition state was obtained from a transition state search starting from the highest image of the NEB path. The final configurations of transition states were these identified as TS1 and TS2 (see the Supplementary Materials).
