# Supplementary material for: Advances in heavy alkaline earth chemistry provide insight into complexation of weakly polarizing Ra2+, Ba2+, and Sr2+ cations
Source: Sci Adv. 2024 Jan 5;10(1):eadj8765. doi: 10.1126/sciadv.adj8765 (PMC10776001; doi:10.1126/sciadv.adj8765)
Supplement: Supplementary file 1 — Supplementary Text Figs. S1 to S15 Tables S1 to S4 Legends for data S1 and S2 [file sciadv.adj8765_sm.pdf]

Science Advances
AAAS

Supplementary Materials for

Advances in heavy alkaline earth chemistry provide insight into complexation of weakly polarizing Ra^{2+}, Ba^{2+}, and Sr^{2+} cations

J. Connor Gilhula *et al.*

Corresponding author: Sara L. Adelman, sadelman@lanl.gov; Enrique R. Batista, erb@lanl.gov; Stosh A. Kozimor, stosh@lanl.gov; Nikki A. Thiele, thielena@ornl.gov; Ping Yang, pyang@lanl.gov.

Sci. Adv. **10**, eadj8765 (2024)
DOI: 10.1126/sciadv.adj8765

This PDF file includes:

Supplementary Text
Figs. S1 to S15
Tables S1 to S4
Legends for data S1 and S2

Other Supplementary Material for this manuscript includes the following:

Data S1 and S2

Supplementary Text

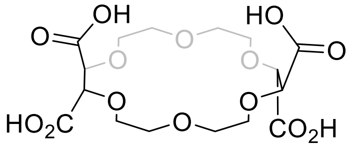

H_4COCO

H_4COCO is a commercial compound obtained from Sigma-Aldrich. NMR characterization data are included here for completeness (*41*).

^{1}H NMR (400 MHz, CD_3CN) δ 4.40 (s, 4H), 3.74 – 3.54 (m, 16H).

^{13}C NMR (101 MHz, CD_3CN) δ 171.3, 80.8, 71.6, 70.5.

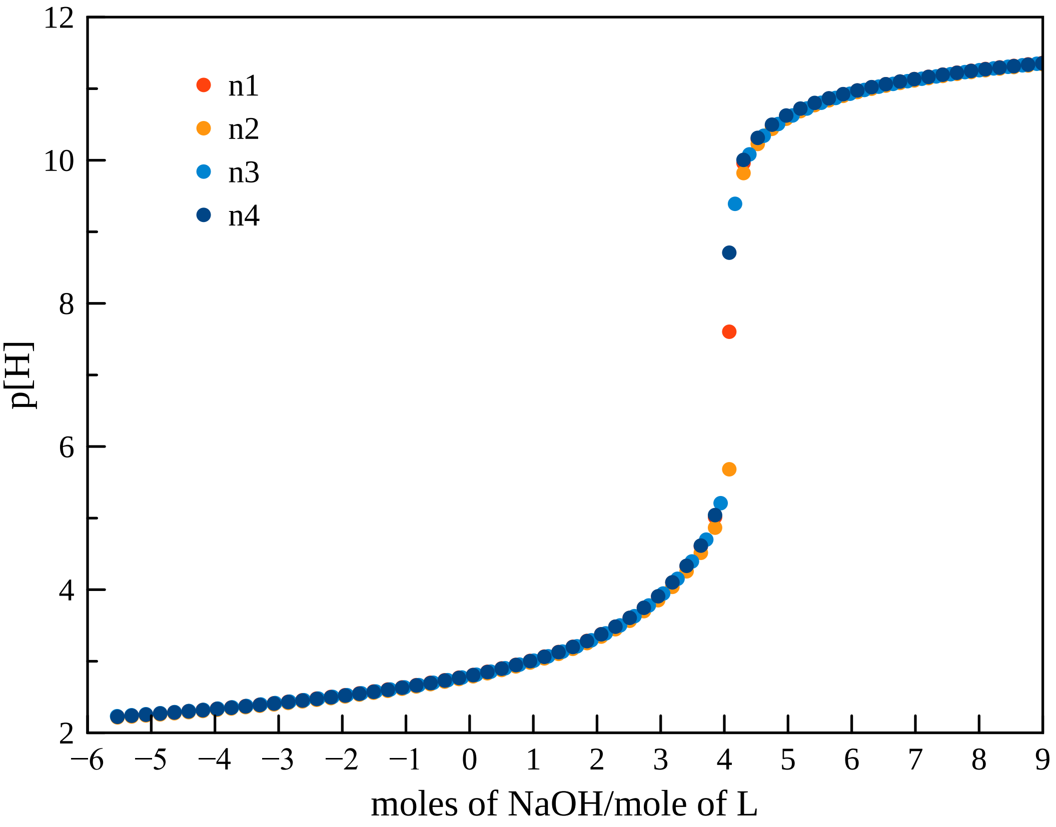

Fig. S1.

Overlay of the potentiometric titration curves of $\mathbf{H_4COCO}$ (1 mM) from each replicate (n = 4). I = 0.2 M NaCl, 25 °C. Note that 1 mL of 0.1 M HCl was added for each replicate to ensure that every titration started at pH 2.3; this "extra" acid is neutralized at the beginning of the titration and appears as negative moles of NaOH/mole of $\mathbf{H_4COCO}$.

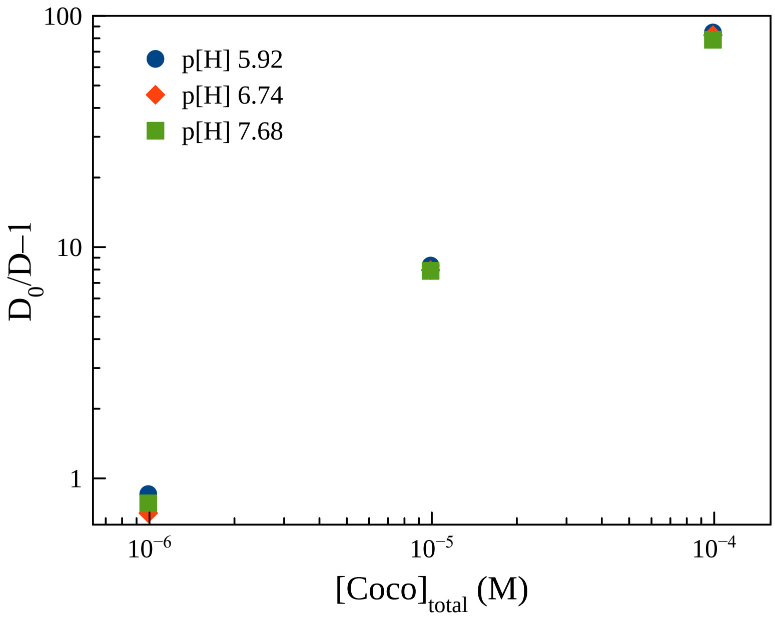

Fig. S2.

Log-log plot of $D_0/D–1$ versus **COCO**$^{4–}$ concentration from preliminary distribution experiments at pH 5.92 (blue circles), pH 6.74 (red diamonds), and pH 7.68 (green squares). The data points from each pH overlap to form a single line, indicating that no protonated complexes of **Ra(COCO)**$^{2–}$ form in aqueous solution over the pH range studied.

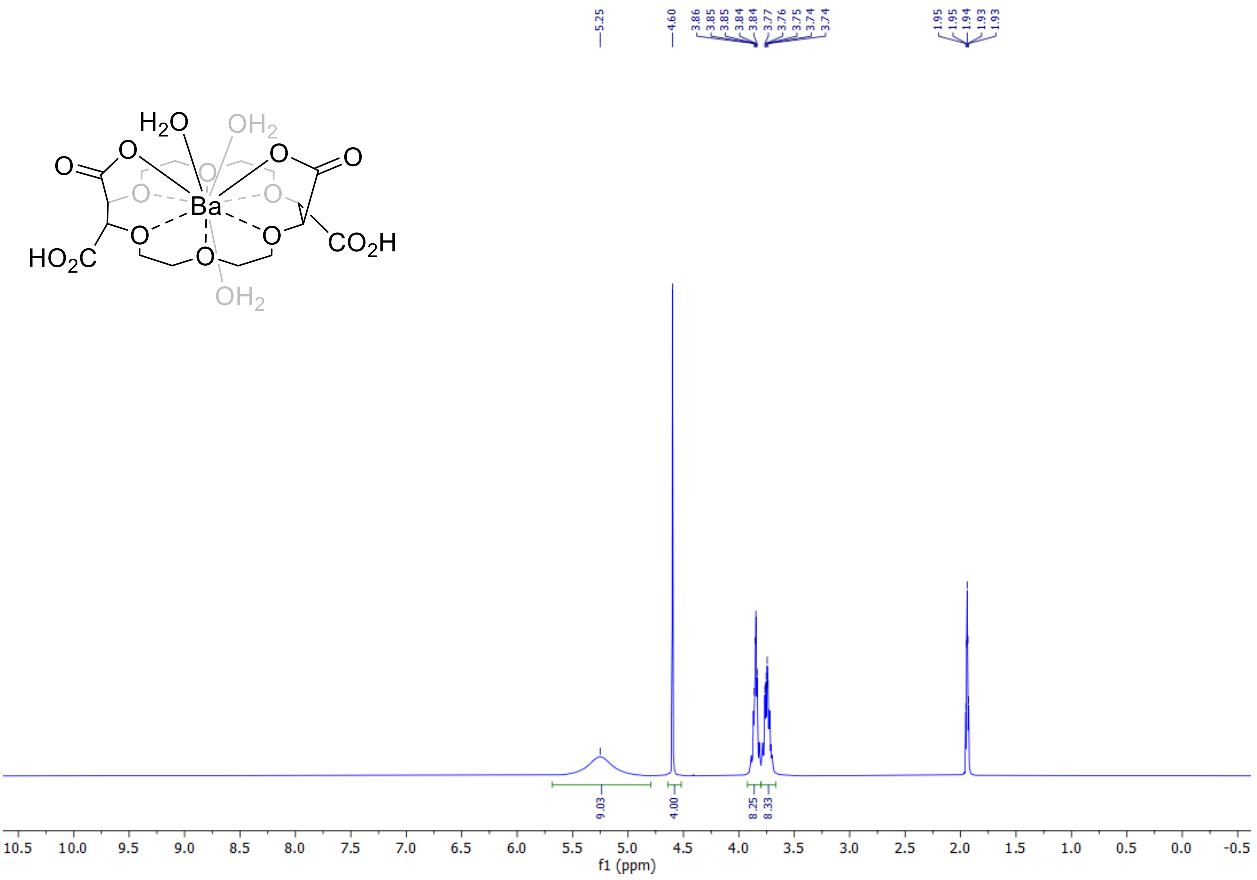

Fig. S3.

^{1}H NMR spectrum of **Ba(H$_2$COCO)** in CD_3CN.

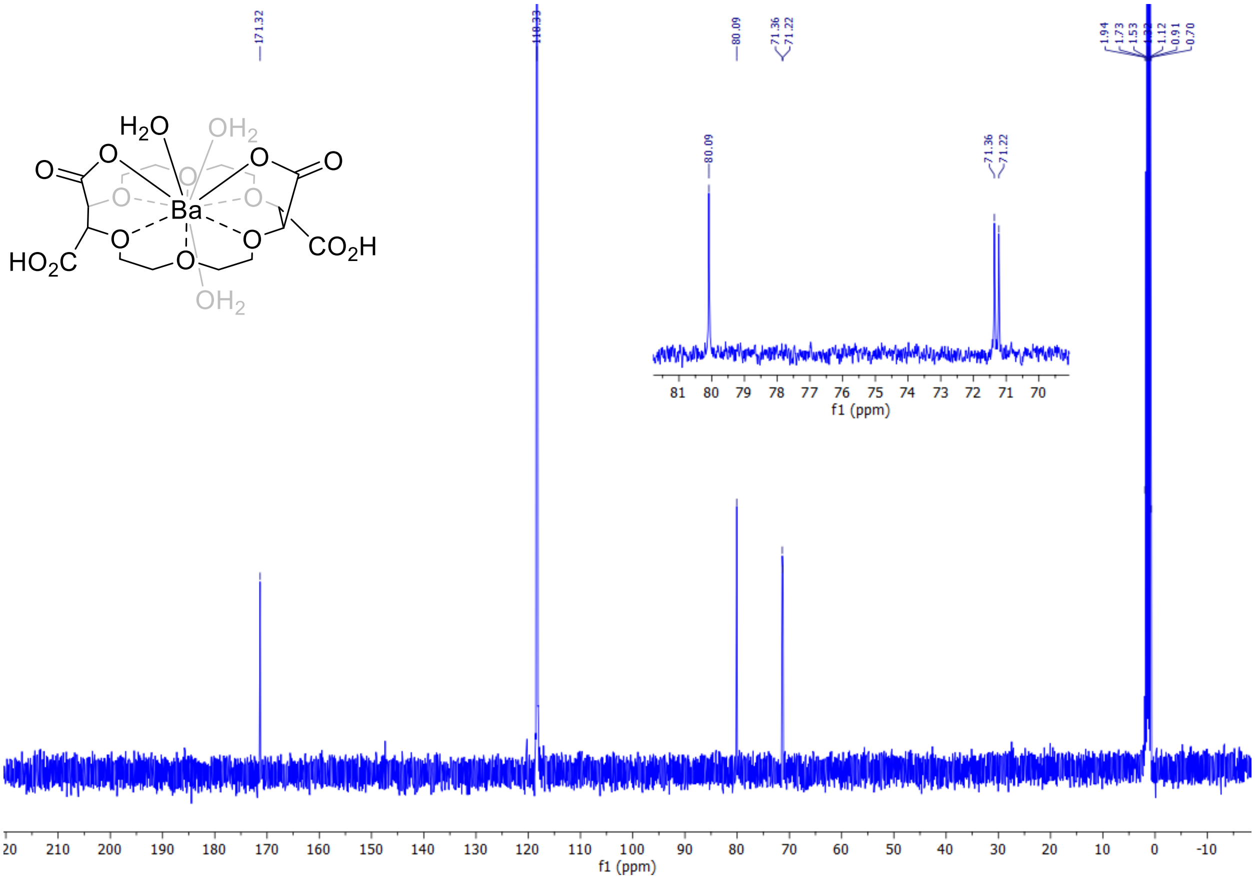

Fig. S4.

$^{13}C\{^{1}H\}$ NMR spectrum of **Ba(H₂COCO)** in CD_3CN.

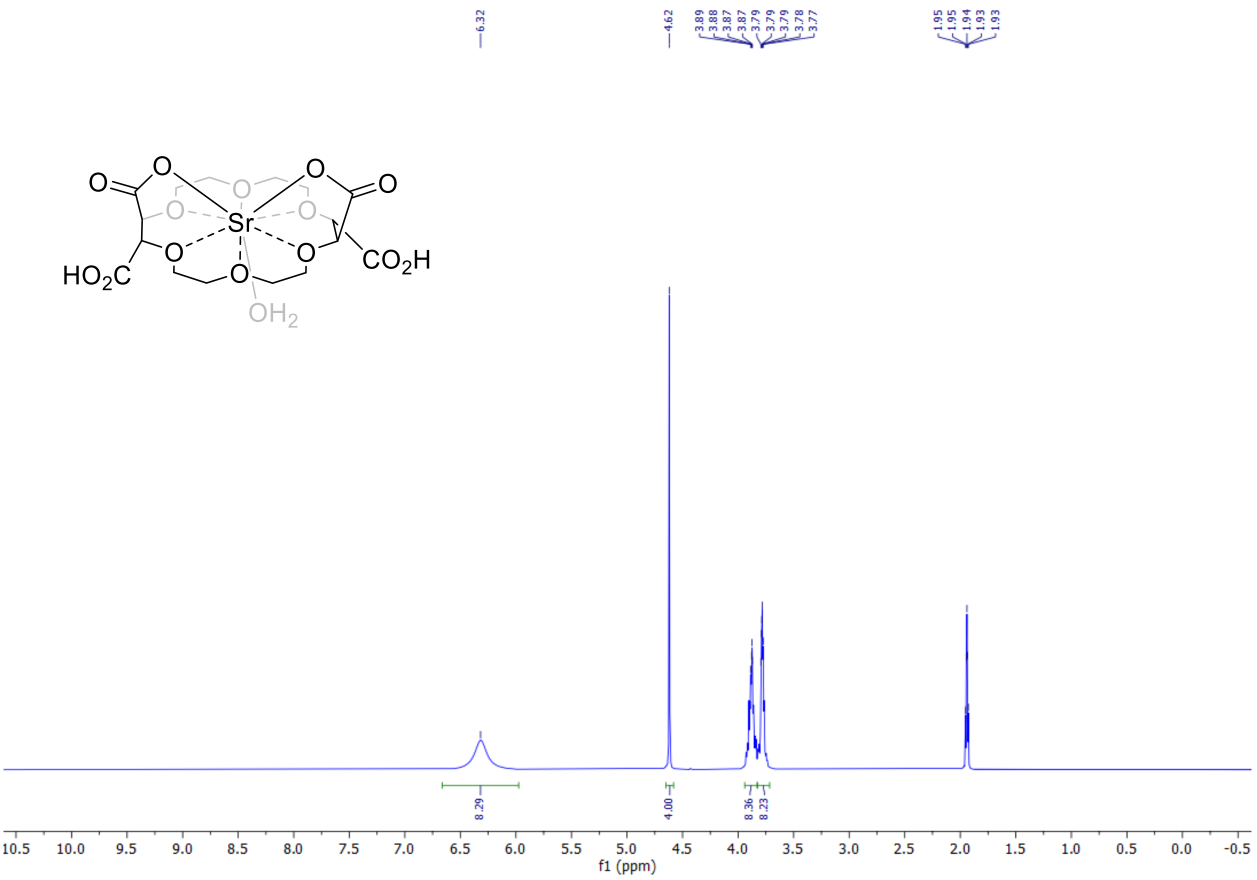

Fig. S5.

^{1}H NMR spectrum of **Sr(H$_2$COCO)** in CD_3CN.

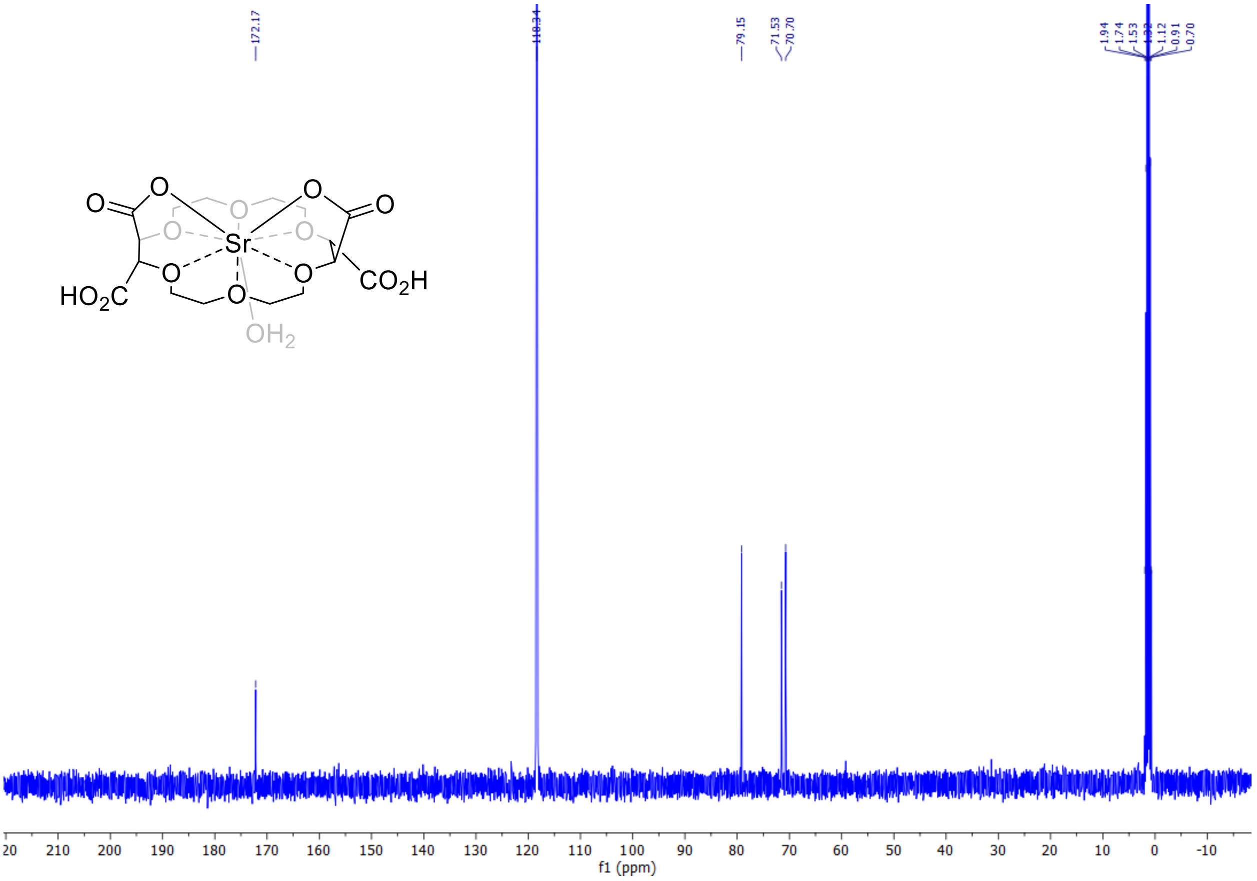

Fig. S6.

$^{13}C\{^{1}H\}$ NMR spectrum of **Sr(H$_2$COCO)** in CD_3CN.

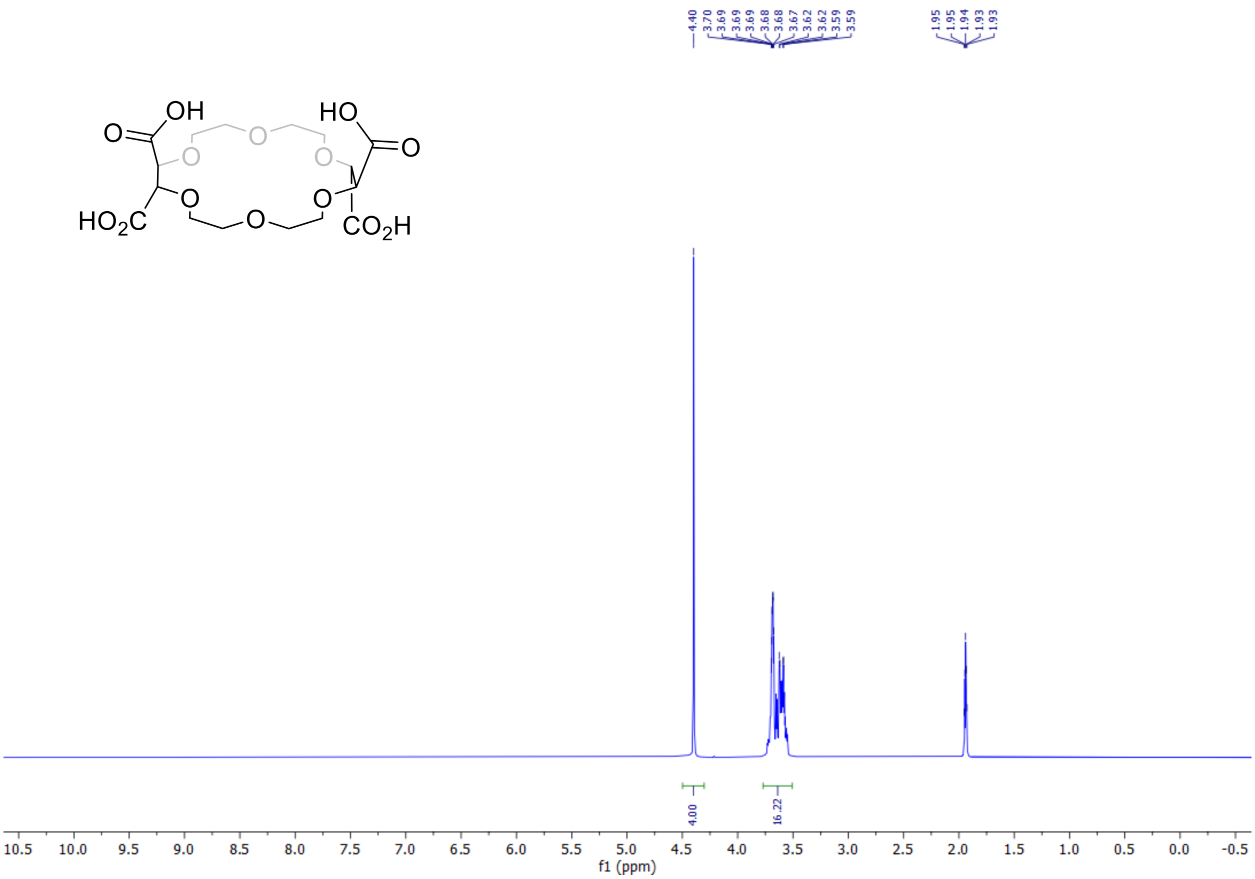

Fig. S7.

^{1}H NMR spectrum of **H_4COCO** in CD_3CN.

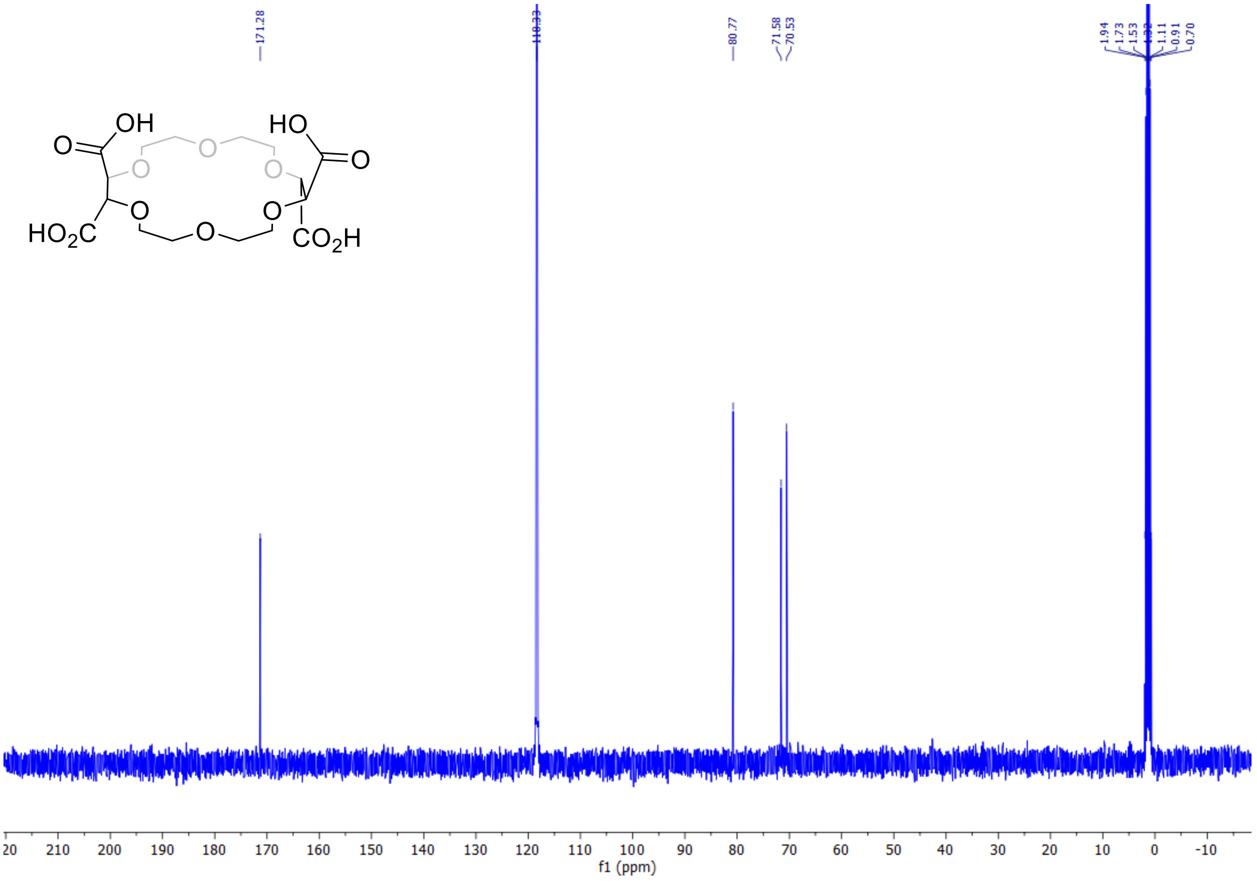

Fig. S8.

$^{13}C\{^{1}H\}$ NMR spectrum of **H_4COCO** in CD_3CN.

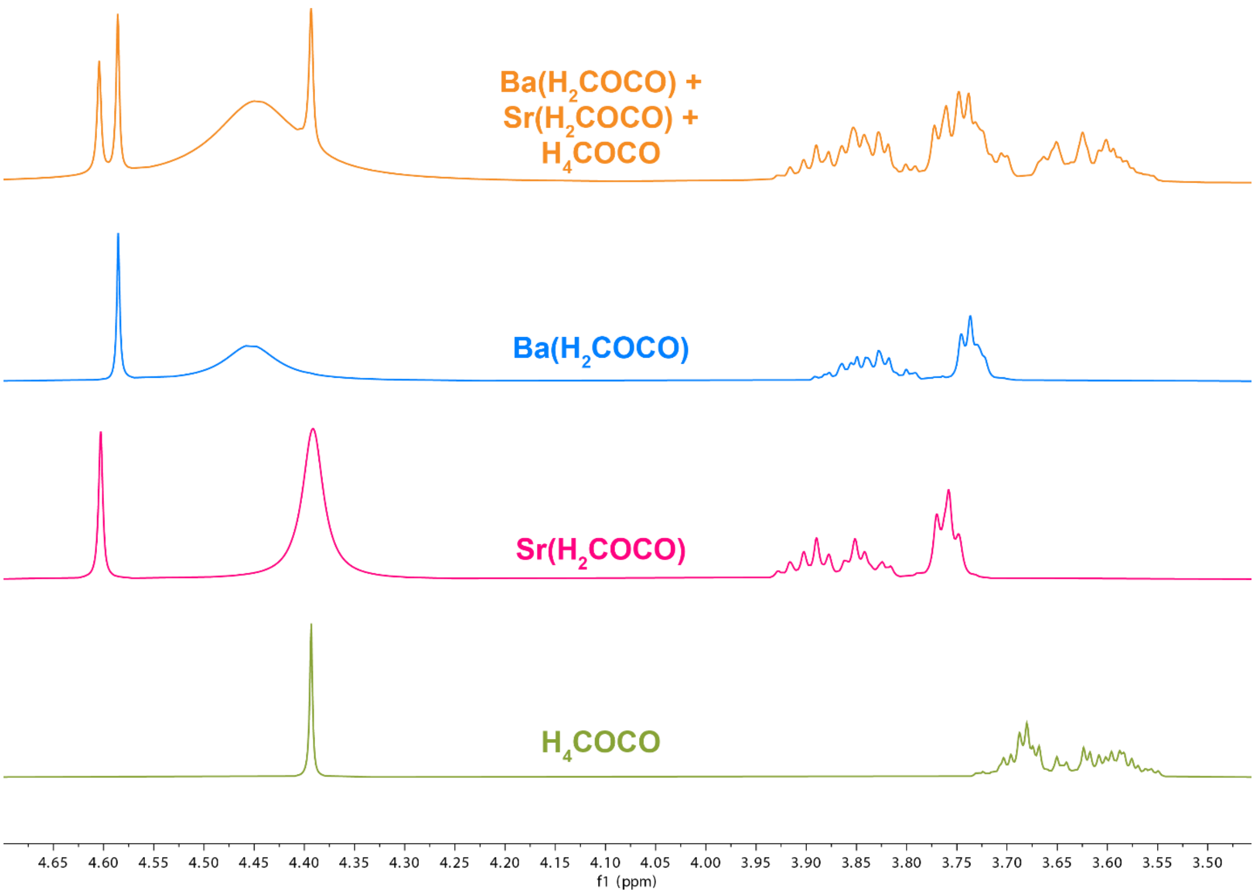

Fig. S9.

^{1}H NMR spectra in CD_3CN of (from bottom to top) **H_4COCO**, **Sr(H_2COCO)**, **Ba(H_2COCO)**, and a mixture of all three species.

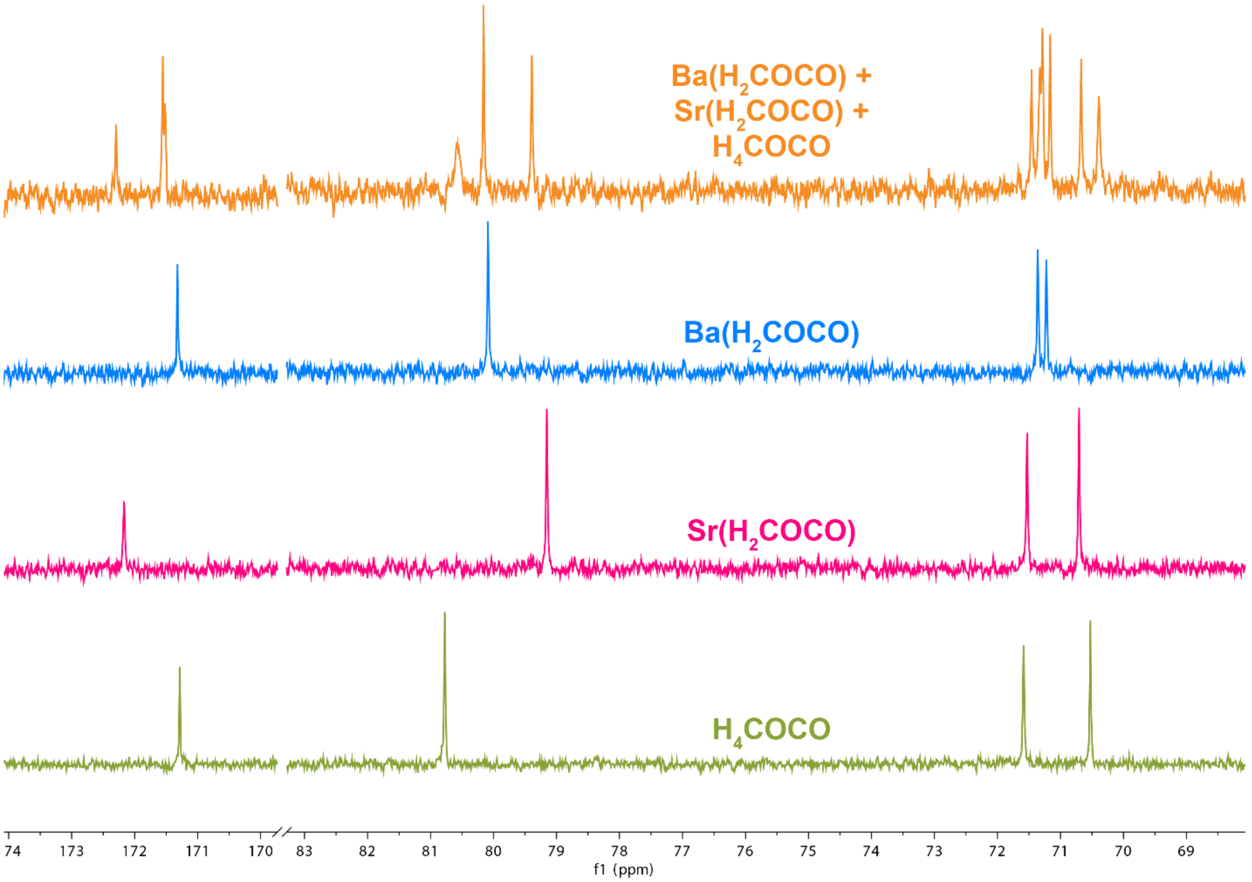

Fig. S10.

$^{13}C\{^{1}H\}$ NMR spectra in CD_3CN of (from bottom to top) **H_4COCO**, **Sr(H_2COCO)**, **Ba(H_2COCO)**, and a mixture of all three species. Note that the mixed spectrum does not perfectly align with its constituent species because the complexes interact by aggregation via the carboxylic acid functions.

Target Compound Screening Report

Results Acquired by The University of Texas at Austin Mass Spectrometry Facility

**Data File**	MSF23-BaCOCO_hrESIpos1.d	**Sample Name**	BaCOCO	**Comment**	BaCOCO
**Position**	P1-B5	**Instrument Name**	Instrument 1	**User Name**	
**Acq Method**	FIA_pos.m	**Acquired Time**	2/6/2023 12:25:26 PM	**DA Method**	KS.m

MS Zoomed Spectrum

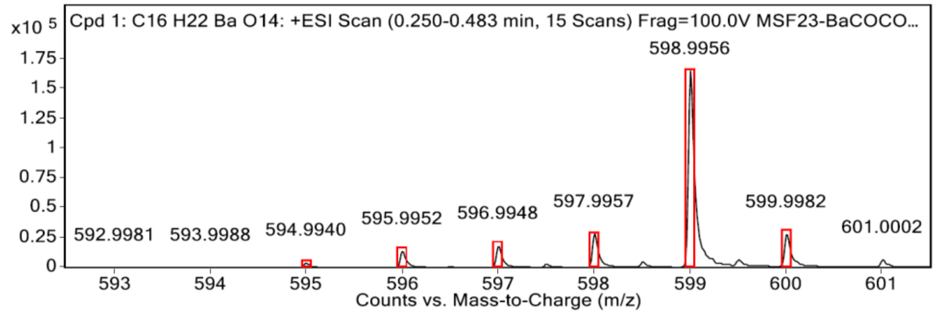

MS Spectrum Peak List

Obs. m/z	Calc. m/z	Charge	Abundance	Formula	Ion Species	Tgt Mass Error (ppm)
592.9981	592.9955	1	215	C16H22BaO14	(M+Na)+	-4.31
594.9940	594.9947	1	4466	C16H22BaO14	(M+Na)+	1.1
595.9952	595.9960	1	14026	C16H22BaO14	(M+Na)+	1.3
596.9948	596.9954	1	18536	C16H22BaO14	(M+Na)+	1.07
597.9957	597.9964	1	28421	C16H22BaO14	(M+Na)+	1.06
598.9956	598.9956	1	165698	C16H22BaO14	(M+Na)+	-0.02
599.9982	599.9989	1	28188	C16H22BaO14	(M+Na)+	1.23
601.0002	601.0007	1	6785	C16H22BaO14	(M+Na)+	0.72
602.0094	602.0035	1	1056	C16H22BaO14	(M+Na)+	-9.87
603.0206	603.0055	1	261	C16H22BaO14	(M+Na)+	-25.01

--- End Of Report ---

Fig. S11.

HRMS report for **Ba(H_2COCO)**.

Target Compound Screening Report

Results Acquired by The University of Texas at Austin Mass Spectrometry Facility

**Data File**	MSF23-SrCOCO_hrESIpos2.d	**Sample Name**	SrCOCO	**Comment**	SrCOCO
**Position**	P1-B6	**Instrument Name**	Instrument 1	**User Name**	
**Acq Method**	FIA_pos.m	**Acquired Time**	2/6/2023 12:32:36 PM	**DA Method**	KS.m

MS Zoomed Spectrum

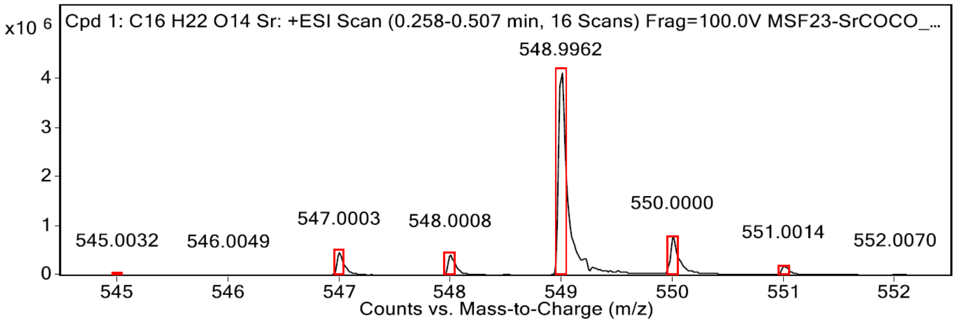

MS Spectrum Peak List

Obs. m/z	Calc. m/z	Charge	Abundance	Formula	Ion Species	Tgt Mass Error (ppm)
545.0032	545.0036	1	25943	C16H22O14Sr	(M+Na)+	0.66
547.0003	546.9995	1	472424	C16H22O14Sr	(M+Na)+	-1.61
548.0008	547.9998	1	425737	C16H22O14Sr	(M+Na)+	-1.71
548.9962	548.9959	1	4151111	C16H22O14Sr	(M+Na)+	-0.43
550.0000	549.9993	1	791977	C16H22O14Sr	(M+Na)+	-1.14
551.0014	551.0010	1	198046	C16H22O14Sr	(M+Na)+	-0.59
552.0070	552.0039	1	28650	C16H22O14Sr	(M+Na)+	-5.59
553.0249	553.0059	1	6649	C16H22O14Sr	(M+Na)+	-34.45
554.0076	554.0085	1	5338	C16H22O14Sr	(M+Na)+	1.6
555.0437	555.0106	1	38397	C16H22O14Sr	(M+Na)+	-59.57

--- End Of Report ---

Fig. S12.

HRMS report for **Sr(H_2COCO)**.

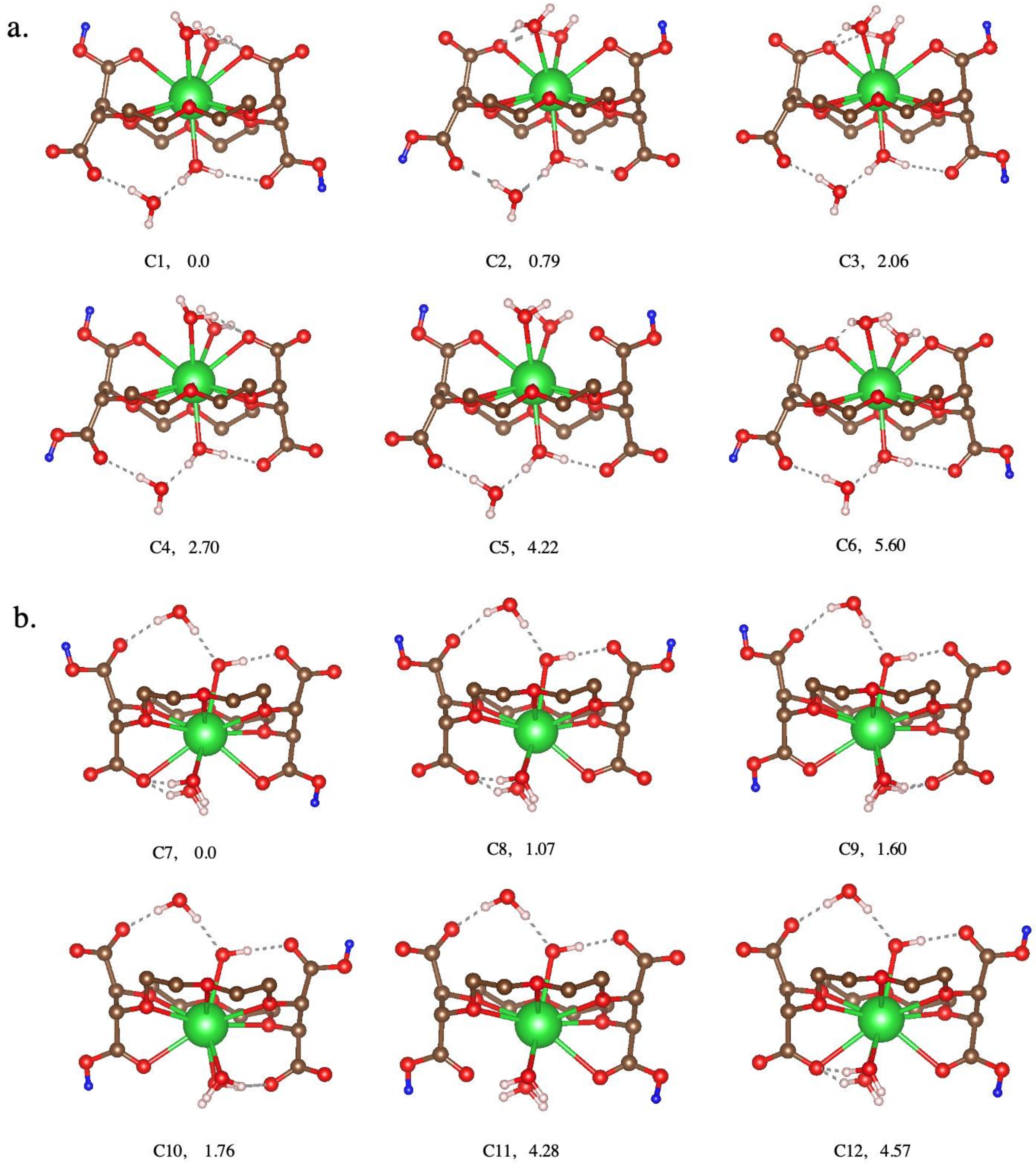

Fig. S13.

The two protons (indicated by blue spheres) on carboxylic acid substituents in various sites with respect to the Ba^{2+} at different sides of the crown ether in the DFT-optimized structure of **Ba(H_2COCO)**. The dashed lines in the structures represent hydrogen bonds. The C1 and C7 configurations correspond to the most stable ones when Ba^{2+} migrates from one side (a) to the other side (b). The corresponding relative electronic energy E for each configuration is provided as black numbers.

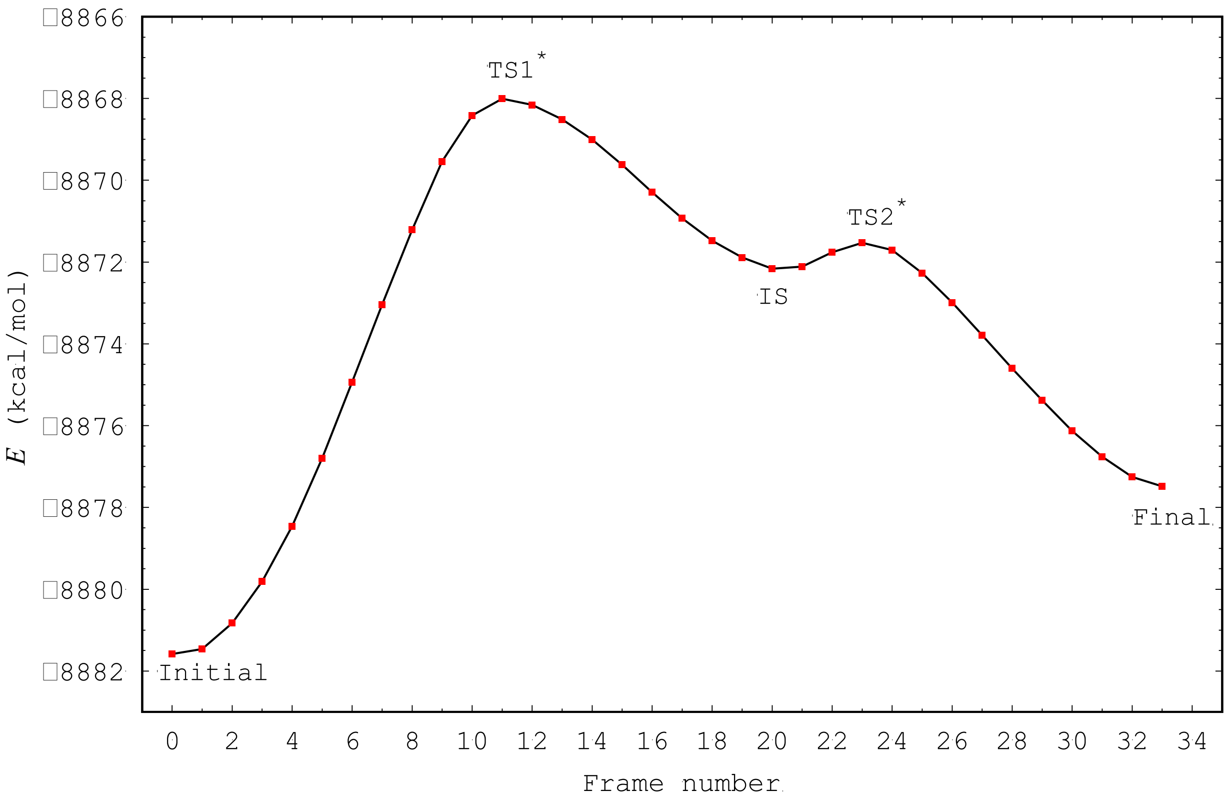

Fig. S14.

The reaction coordinate obtained from NEB calculations connecting initial, intermediate, and final systems.

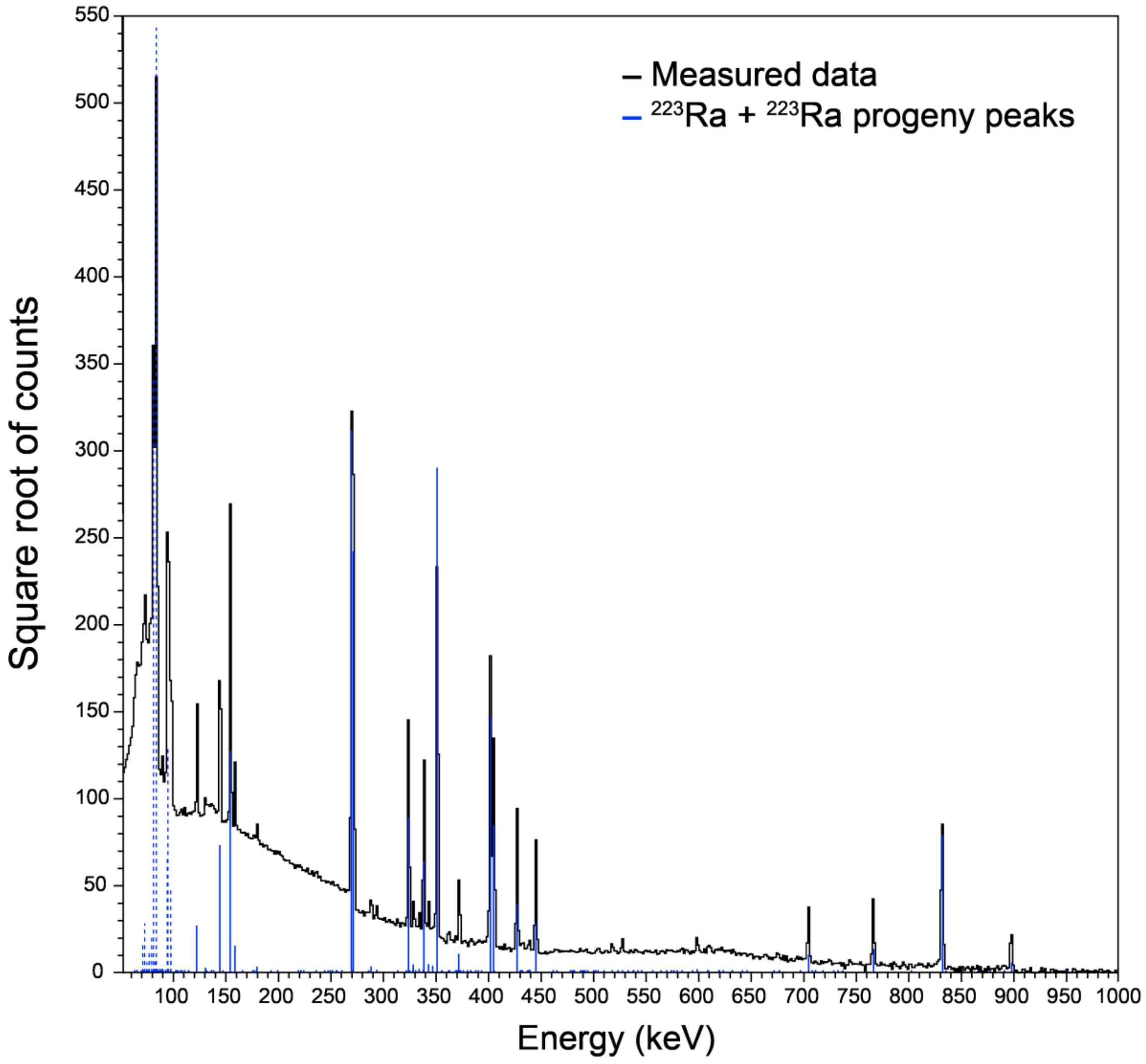

Fig. S15.

Representative γ spectrum of isolated ^{223}Ra (black). Blue lines indicate expected γ-ray emissions for ^{223}Ra and its radioactive progenies.

Table S1.
Protonation constants of **H_4COCO** determined at 25 °C and I = 0.2 M NaCl.

	**$COCO^{4-}$**
log K_{a1}	4.44 ± 0.04
log K_{a2}	3.33 ± 0.02
log K_{a3}	2.66 ± 0.02
log K_{a4}	1.90 ± 0.14

Table S2.

Crystal data and structure refinement for **Ba(H_2COCO)**.

CCDC Deposition No.	2237641
Empirical formula	$C_{16}H_{34}BaO_{20}$
Formula weight	683.77
Temperature/K	100.00
Crystal system	orthorhombic
Space group	$P2_12_12_1$
a/Å	12.6139(17)
b/Å	13.8026(18)
c/Å	29.256(4)
α/°	90
β/°	90
γ/°	90
Volume/Å^3	5093.6(12)
Z	8
ρ_{calc}g/cm^3	1.783
μ/mm^{-1}	1.650
F(000)	2768.0
Crystal size/mm^3	0.25 × 0.1 × 0.1
Radiation	MoKα (λ = 0.71073)
2Θ range for data collection/°	2.784 to 68.276
Index ranges	$-19 \leq h \leq 19$, $-21 \leq k \leq 21$, $-46 \leq l \leq 46$
Reflections collected	197951
Independent reflections	20897 [R_{int} = 0.0613, R_{sigma} = 0.0348]
Data/restraints/parameters	20897/0/763
Goodness-of-fit on F^2	1.079
Final R indexes [I>=2σ (I)]	R_1 = 0.0349, wR_2 = 0.0731
Final R indexes [all data]	R_1 = 0.0514, wR_2 = 0.0788
Largest diff. peak/hole / e Å^{-3}	2.40/-0.72
Flack parameter	-0.007(4)

Table S3.

Crystal data and structure refinement for **Sr(H_2COCO)**.

CCDC Deposition No.	2237642
Empirical formula	$C_{16}H_{36}O_{21}Sr$
Formula weight	652.07
Temperature/K	100.00
Crystal system	monoclinic
Space group	C2
a/Å	12.3344(3)
b/Å	9.1933(2)
c/Å	11.5132(3)
α/°	90
β/°	90.1630(10)
γ/°	90
Volume/Å^3	1305.52(5)
Z	2
ρ_{calc}g/cm^3	1.659
μ/mm^{-1}	2.158
F(000)	676.0
Crystal size/mm^3	0.1 × 0.1 × 0.1
Radiation	MoKα (λ = 0.71073)
2Θ range for data collection/°	5.526 to 55.044
Index ranges	-16 ≤ h ≤ 16, -11 ≤ k ≤ 11, -14 ≤ l ≤ 14
Reflections collected	13387
Independent reflections	2890 [R_{int} = 0.0403, R_{sigma} = 0.0380]
Data/restraints/parameters	2890/2/186
Goodness-of-fit on F^2	1.156
Final R indexes [I>=2σ (I)]	R_1 = 0.0318, wR_2 = 0.0885
Final R indexes [all data]	R_1 = 0.0321, wR_2 = 0.0886
Largest diff. peak/hole / e Å^{-3}	2.85/-0.75
Flack parameter	0.008(3)

Table S4.

The DFT/PBE optimized Ba–$O_{acetate}$, Ba–O_{crown}, and Ba–O_{H_2O} bond distances (Å) and total energies *E* of initial system, transition system 1 (TS1), intermediate system (IS), transition system 2 (TS2), and final system. The electronic energy *E*, Gibbs free energy *G* (298.15 K), enthalpy *H*, entropy *S*, and zero-point energy (ZPE) are included.

	Initial	TS1	IS	TS2	Final
Ba–$O_{acetate}$	2.927; 2.920<br>4.574; 5.163	3.992; 4.393<br>4.050; 4.026	4.262; 4.426<br>3.928; 3.886	4.341; 4.629<br>3.645; 3.639	3.044; 3.128<br>4.501; 5.023
Ba–O_{crown}	2.961; 2.847;<br>2.922; 2.924;<br>2.885; 2.914	2.862; 2.726;<br>2.766; 2.817;<br>2.744; 2.782	2.853; 2.723;<br>2.747; 2.821;<br>2.725; 2.779	2.873; 2.722;<br>2.757; 2.832;<br>2.731; 2.789	3.002; 2.773;<br>2.836; 2.894;<br>2.785; 2.920
Ba–O_{H_2O}	2.980; 2.967<br>2.754; 4.766	2.990; 2.873<br>2.850; 3.546	3.012; 2.907<br>2.936; 2.984	2.941; 2.917<br>2.936; 3.596	2.877; 2.881<br>2.876; 5.033
E(kcal/mol)	− 8881.59	− 8871.26	− 8872.13	− 8871.64	− 8877.49
G(kcal/mol)	− 8626.62	− 8614.61	− 8616.88	− 8615.93	− 8622.37
H(kcal/mol)	− 8550.53	− 8540.94	− 8540.71	− 8540.84	− 8546.52
S(cal/mol•K^{-1})	255.19	247.07	255.48	251.88	254.38
ZPE (kcal/mol)	303.34	303.65	303.78	303.65	303.65

Data S1. Crystallographic information file for Ba(H2COCO).
Data from X-ray diffraction experiments on crystals of **Ba(H_2COCO)**.

Data S2. Crystallographic information file for Sr(H2COCO).
Data from X-ray diffraction experiments on crystals of **Sr(H_2COCO)**
